# High-Throughput Synthesis, Purification, and Application of Alkyne-Functionalized Discrete Oligomers

**DOI:** 10.1021/jacs.4c00751

**Published:** 2024-03-15

**Authors:** Junfeng Chen, Vittal Bhat, Craig J. Hawker

**Affiliations:** † Materials Department, Materials Research Laboratory, and Department of Chemistry and Biochemistry, University of California, Santa Barbara, California 93106, United States; ‡ Department of Chemistry, University of North Carolina, Chapel Hill, North Carolina 27599, United States

## Abstract

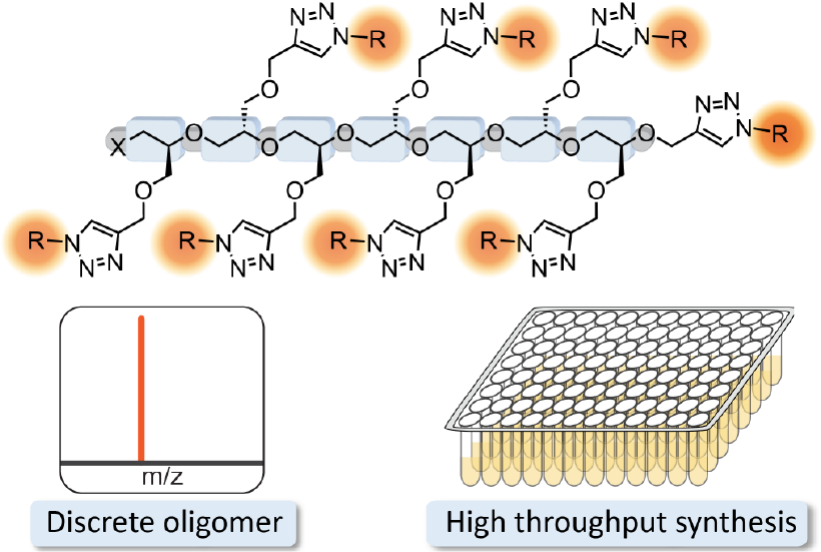

The development of synthetic oligomers as discrete single molecular entities with accurate control over the number and nature of functional groups along the backbone has enabled a variety of new research opportunities. From fundamental studies of self-assembly in materials science to understanding efficacy and safety profiles in biology and pharmaceuticals, future directions are significantly impacted by the availability of discrete, multifunctional oligomers. However, the preparation of diverse libraries of discrete and stereospecific oligomers remains a significant challenge. We report a novel strategy for accelerating the synthesis and isolation of discrete oligomers in a high-throughput manner based on click chemistry and simplified bead-based purification. The resulting synthetic platform allows libraries of discrete polyether oligomers to be prepared and the impact of variables such as chain length, number, and nature of side chain functionalities and molecular dispersity on antibacterial behavior examined. Significantly, discrete oligomers were shown to exhibit enhanced activity with lower toxicity compared with traditional disperse samples. This work provides a practical and scalable methodology for nonexperts to prepare libraries of multifunctional discrete oligomers and demonstrates the advantages of discrete materials in biological applications.

## Introduction

The convergence of organic synthesis and polymer chemistry has enabled the design and study of oligomers and polymers with unprecedented levels of control over the molecular structure. By bringing the molecular precision associated with small molecules to macromolecular systems, strategies for the scalable preparation of discrete, multifunctional oligomers allow key structural parameters such as the number/arrangement of functional units and molar mass/degree of polymerization to be addressed.^1−5^ The importance of developing robust strategies for these discrete systems is further exemplified by the significant potential that synthetic oligomers have shown in various biological and pharmaceutical applications. For example, multifunctional oligomers can be employed as delivery vehicles for drugs, proteins, or nucleic acids to achieve specific targeting effects and enhanced efficiency.^6−8^ Additionally, emerging biotechnology strategies such as the lysosomal targeting chimera (LYTAC) platform,^9^ synthetic antimicrobial materials,^10,11^ and gene therapies utilize synthetic macromolecules directly.^12^ However, unlike small molecules or biomacromolecules with precisely defined structures, synthetic macromolecules are typically dispersed in molar mass and the number of functional units along the polymer backbone. As a result, each molecular entity within this disperse mixture can exhibit different therapeutic effectiveness and toxicity which precludes a complete understanding of structure–activity profiles.^13−15^ This impedes future application of synthetic oligomers and renders scalable libraries inaccessible to nonexperts and the broader biology/material science communities.^16^ A growing need therefore exists for user-friendly approaches to the preparation of discrete oligomer libraries that are accessible to the general community in standard high-throughput formats.

To address these challenges,^17^ discrete oligomers can be prepared through stepwise synthetic strategies based on efficient coupling reactions^18−25^ or through novel biotechnologies that utilize precise biomacromolecules as templates.^26,27^ To further increase the availability of discrete materials, our group has developed alternative, user-friendly strategies for the scalable synthesis of discrete oligomers,^28^ based on combining controlled polymerization^29,30^ with accelerated chromatographic separation. This two-step process allows disperse parent mixtures to be separated at scale into discrete oligomers with control over the degree of polymerization (DP), chain ends, and overall molecular structure. Utilizing this platform, libraries of discrete oligomers with targeted DP have been prepared from a single synthesis step with the resulting discrete materials exhibiting chain-length-dependent luminescence, morphological, and self-assembly properties.^31−33^

**Figure 1:**
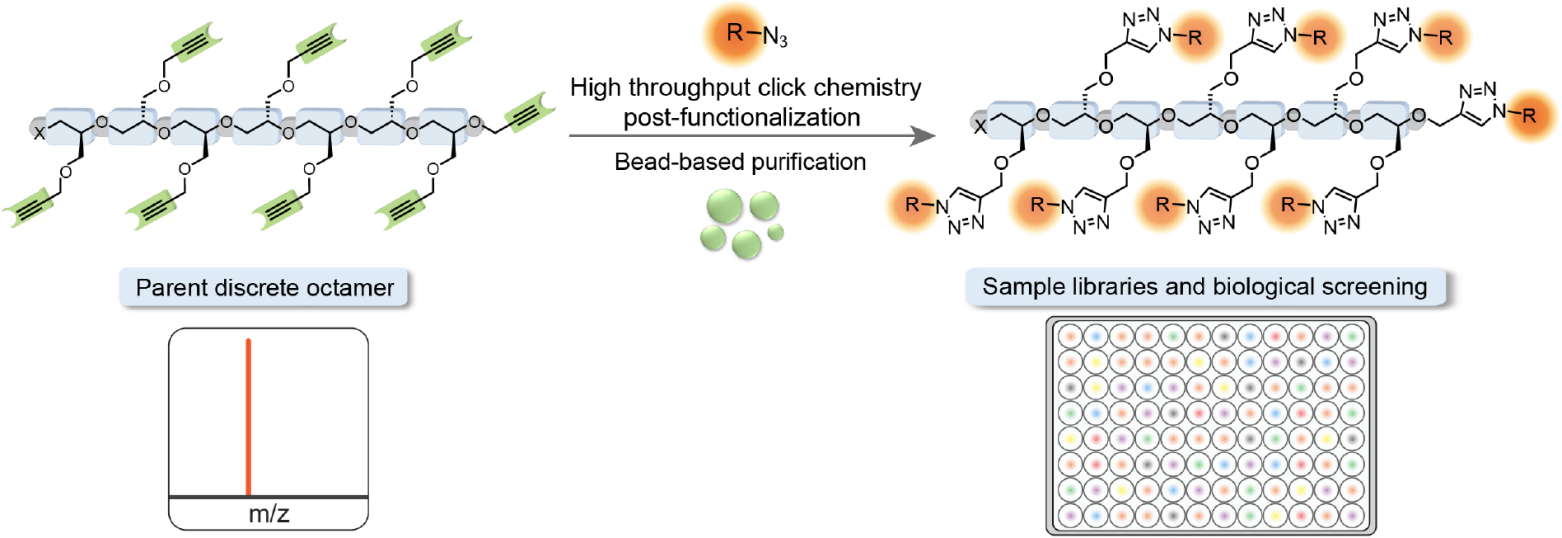
Schematic summary of the high-throughput synthesis and purification strategy for the preparation of discrete oligomer libraries.

Driven by the promise of widely available discrete materials,^34−36^ a high-throughput strategy for preparing functionalized libraries based on the combination of ring opening polymerization (ROP) and alkyne–azide click chemistry is described (Figure 1). From propargyl glycidyl ether, controlled ring opening polymerization is employed to prepare stereoregular oligomer mixtures consisting of a polyether backbone and multiple alkyne side chain/chain end groups. This disperse oligomer mixture is then separated into discrete fractions using automated chromatography with accurate control over oligomer length and structural purity. Subsequently, copper-mediated alkyne–azide click reactions (CuAAC) are performed in a simple, 96-well array format that allows for facile postfunctionalization and purification of discrete oligomers. Through reaction with a range of different azides, control over the number and nature of functional groups is possible leading to a series of discrete oligomer libraries.^37,38^ To further enable the use of this strategy by nonexperts, we report the development of a facile high-throughput purification method utilizing customized purification beads for removal of impurities and excess reagents from these multifunctional libraries.

## Results and Discussion

### Design and Synthesis of the Alkyne Containing Discrete Oligomers

**Scheme 1:**
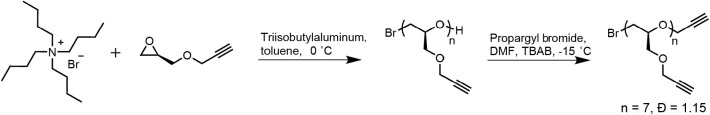
Ring Opening Polymerization of Glycidyl Propargyl Ether

Alkyne-functionalized oligomers were prepared through the living ring opening polymerization of (R)-glycidyl propargyl ether (Scheme 1).^39,40^ In designing this system, two major considerations were the presence of multiple alkyne groups, which allows for facile CuAAC post-functionalization and generation of oligomeric libraries. Second, the use of glycidyl monomers leads to polyether backbones, which are flexible, biocompatible, and hydrolytically stable in biological environments.^41^ Moreover, anionic ring opening polymerization preserves the initial monomer stereochemistry, opening the possibility of preparing discrete stereospecific oligomers. Following polymerization, the hydroxyl chain end is modified by reaction with propargyl bromide to introduce an additional alkyne unit at the chain end, thereby capping the polar hydroxyl chain end and improving the efficiency of the chromatography separation. As a result, each molecule contains DP + 1 alkyne groups. Using (R)-glycidyl propargyl ether and tetra-*n*-butyl ammonium bromide as the initiator, ring opening polymerization is easily scaled to give 20+ g of oligomer with control over molar mass via varying the initiator/monomer ratio. The dispersity present in the as-formed parent oligomers can be observed by matrix-assisted laser desorption/ionization (MALDI) mass spectrometry and size exclusion chromatography (SEC). An illustrative sample has a dispersity *Đ* = 1.15 and an average degree of polymerization (DP) of 7.

**Figure 2:**
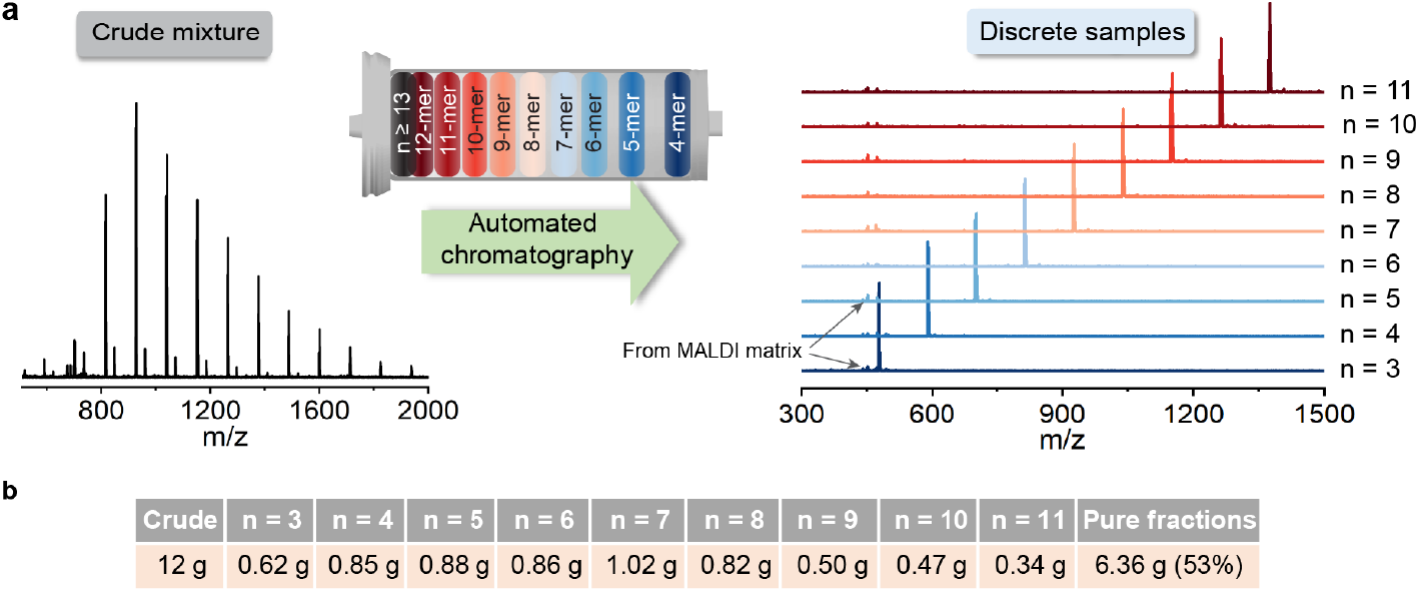
Separation of discrete alkyne containing oligomers. (a) Automated chromatographic separation of oligomers from *n* = 3 to *n* = 11, and MALDI spectra of the discrete alkyne containing oligomers before and after separation. (b) Sample yields of discrete fractions after chromatographic separation.

Multigram quantities of the crude parent oligomer could then be separated by automated chromatography to give a library of discrete oligomers (Figure 2a). For example, from 12 g of the parent disperse oligomer, elution with hexane and ethyl acetate affords nine oligomer fractions which were shown to be discrete single molecules with 4 to 12 alkyne groups (DPs ranging from 3 to 11). Significantly, these discrete oligomers were obtained in quantities ranging from 400 mg to over 1.0 g (Figure 2b). It is noteworthy that this represents greater than 50% mass recovery from the crude polymerization mixture with mixed fractions and higher molar mass oligomers/polymers, bringing total mass recovery to over 90%.

The structural purity of each alkyne oligomer was determined by using a combination of spectroscopic and chromatographic techniques. To illustrate the discrete nature of the oligomers obtained after chromatographic separation, Figure 2a shows the MALDI mass spectra for the parent poly(glycidyl ether) (*Đ* = 1.15) which reveals a series of molecular ions up to *n* = 20 indicating that even under living polymerization conditions, a wide dispersity of oligomer lengths is obtained. In direct contrast, single *m*/*z* peaks are observed for each fractionated oligomer molecular ion peak from 479 to 1374 amu corresponding to the specific molar mass for the desired discrete oligomers having 4-alkyne to 12-alkyne groups along the backbone. The ability to separate the starting disperse oligomer mixture is also illustrated by SEC where analysis of the purified fractions shows a series of narrow symmetrical peaks with gradually decreasing retention time for each oligomer fraction, illustrating the high purity and increasing degrees of polymerization for each oligomer (Figure S10).

**Figure 3:**
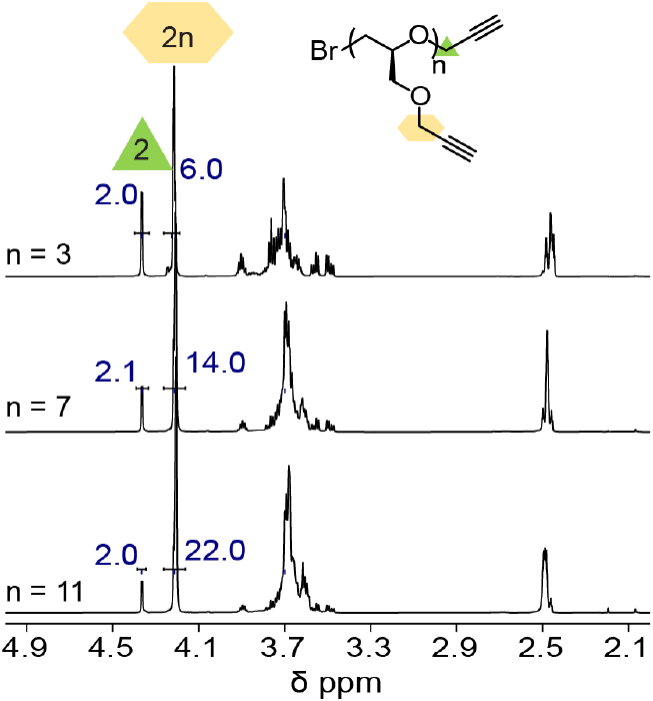
^1^H NMR spectra of discrete alkyne oligomers (DP = 3, DP = 7, DP = 11) showing integration values for the single chain end propargyl unit (triangle) versus side chain propargyl units (hexagon).

**Figure 4:**
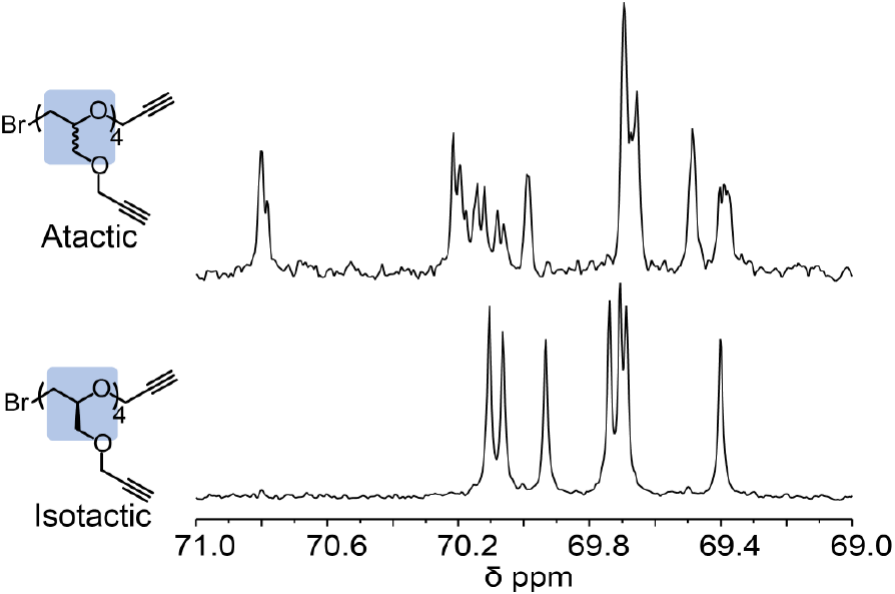
^13^C NMR spectra (69.0 to 71.0 ppm) for the atactic and isotactic alkyne oligomers (*n* = 4) showing stereoregularity for the isotactic derivative.

The discrete nature of the oligomers was also demonstrated by ^1^H NMR and ^13^C NMR spectroscopy, with specific peaks showing systematic changes for unique resonance and integration ratios with increasing DP. As shown in Figure 3, the peak for the methylene group (CH_2_) of the single propargyl chain end unit appears as a singlet at 4.4 ppm, while resonances for the backbone propargyl CH_2_ groups are observed at 4.2 ppm. As oligomer length increases from DP = 3, DP = 7 to DP = 11, the integration ratios systematically increase from 2:6, 2.1:14 to 2:22, which matches the expected values for the discrete structures. Systematic changes in the ^13^C NMR spectra were also observed depending on both the degree of polymerization and the stereochemical purity of the repeat units. For example, atactic tetramers with a stereoirregular backbone lead to multiple chemical shifts for the backbone CH_2_–O units and a complicated set of peaks in the^13^C NMR spectrum. In contrast, for the discrete, stereoregular oligomer with 5 alkyne units, 7 peaks were observed in the backbone region (69.0–71.0 ppm), corresponding to 2*n*-1 backbone methylene groups and indicating only a single stereochemistry for each carbon atom (Figures 4 and S9). Further analysis of ^13^C NMR spectra of the trends between discrete oligomers clearly shows the well-defined structure with stereocenters being preserved and chain end groups being controlled during the anionic polymerization (Figure S9).

### High-Throughput Functionalization and Purification

**Figure 5:**
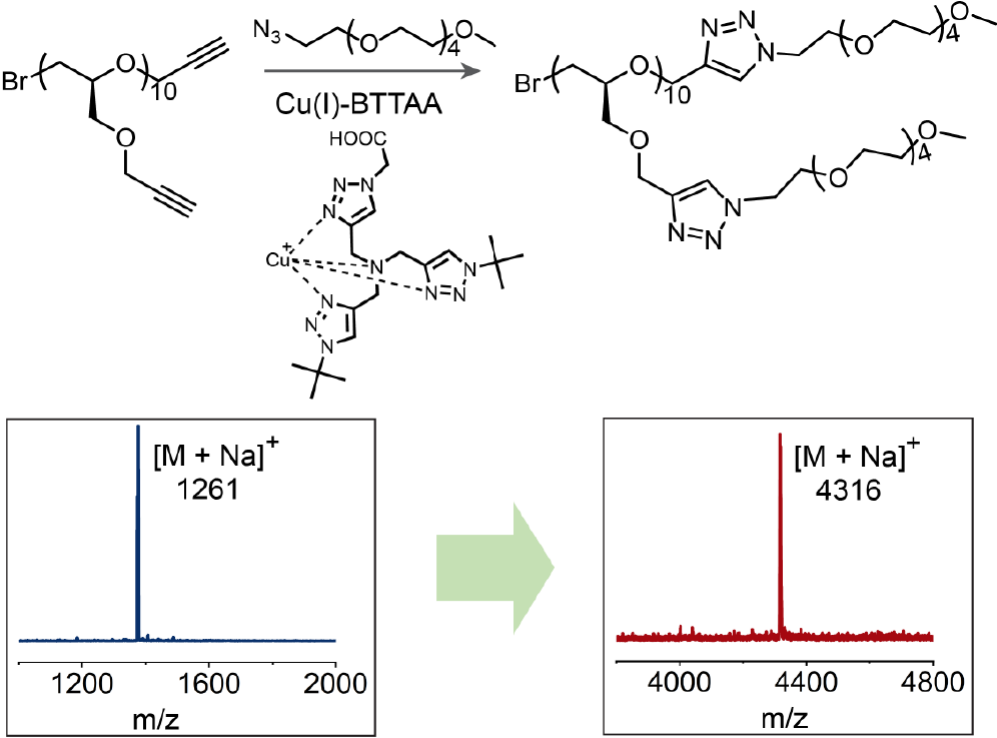
Post-functionalization of alkyne oligomers through CuAAC reaction and MALDI spectra before and after the post-functionalization of the decamer (DP = 10) with 11 reactive alkyne units.

A significant opportunity for these alkyne-functionalized polyether oligomers is as parent platforms for the creation of diverse oligomer libraries through click coupling with different azido derivatives. To illustrate the efficiency of this process, CuAAC click reactions were initially performed between the discrete alkyne oligomers and methoxy penta(ethylene glycol) azide (mPEG_5_-N_3_) with BTTAA-Cu(I) as the catalyst.^42^ As shown in Figure 5b, the starting decamer (DP = 10) with 11-alkyne groups exhibits a single set of molecular ion peaks in MALDI mass spectrometry centered at 1261 amu, which on coupling with mPEG_5_-N_3_ quantitatively gives the fully functionalized derivative with a single set of molecular ions centered at 4316 amu (Figure 5). It is noteworthy that this quantitative functionalization with mPEG_5_-N_3_ is also observed for all of the other discrete oligomeric derivatives studied, from DP = 3 to DP = 11.

With the demonstrated simplicity of CuAAC and the availability of a library of discrete oligomers, this strategy is a powerful tool for parallel and high-throughput reactions^43,44^ leading to the introduction of multiple ionic units. It should be noted that the direct synthesis of these highly functionalized and discrete oligomers would lead to synthesis and purification challenges. In addition, the presence of the copper catalyst and associated ligands can further complicate purification and is detrimental for many applications, for example, toxicity in biological systems. A simple purification process is therefore critical, with conventional methods such as chromatography, recrystallization, distillation, and dialysis being unsuitable for purifying these highly functionalized oligomers and not readily applicable to high-throughput strategies.

**Figure 6:**
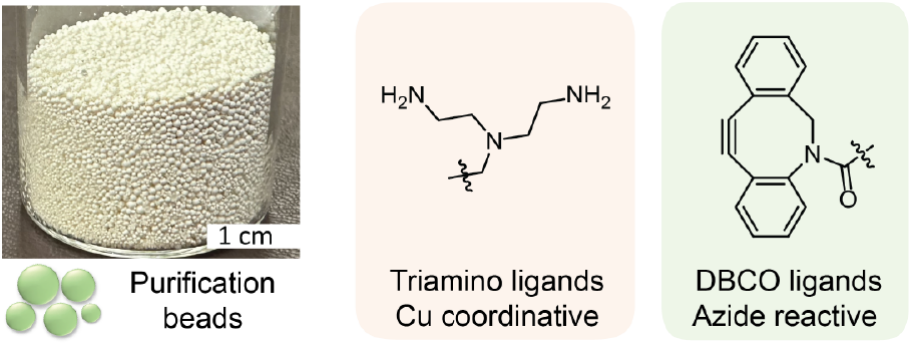
Structure and functionalization of purification beads for Cu-ligation and azide scavenging.

To address these challenges, a simple and robust purification strategy for removing copper ions, BTTAA ligands, and excess azido compounds has been developed. Key to the success of this strategy is the design of novel purification beads with development being guided by two principles: first, the beads must contain coordination ligands capable of preferentially complexing the combined copper-ligand catalyst. However, a significant drawback is that traditional coordinating groups solely bind the Cu ions, making it difficult to find functionalities that will scavenge the full BTTAA-Cu complex, removing both the ligand and Cu ion from the reaction mixture. Thus, we conducted an extensive screening campaign to identify functional groups that are capable of chelating both the BTTAA ligands and Cu ions. Second, the purification beads must exhibit high reactivity toward azido compounds leading to covalent scavenging of excess azide and effective removal during purification of the reaction mixture. To meet these requirements, we developed a series of purification beads through the postfunctionalization of polystyrene beads containing diethylenetriamines units (see Supporting Information for more details). The combination of diethylenetriamine ligands and partial amidation of the primary amine groups with dibenzocyclooctyne (DBCO) units results in efficient removal of Cu ions, BTTAA by chelation and excess azido derivatives by strain-promoted alkyne–azide cycloaddition (SPAAC) coupling at room temperature (Figure 6).^45^

**Figure 7:**
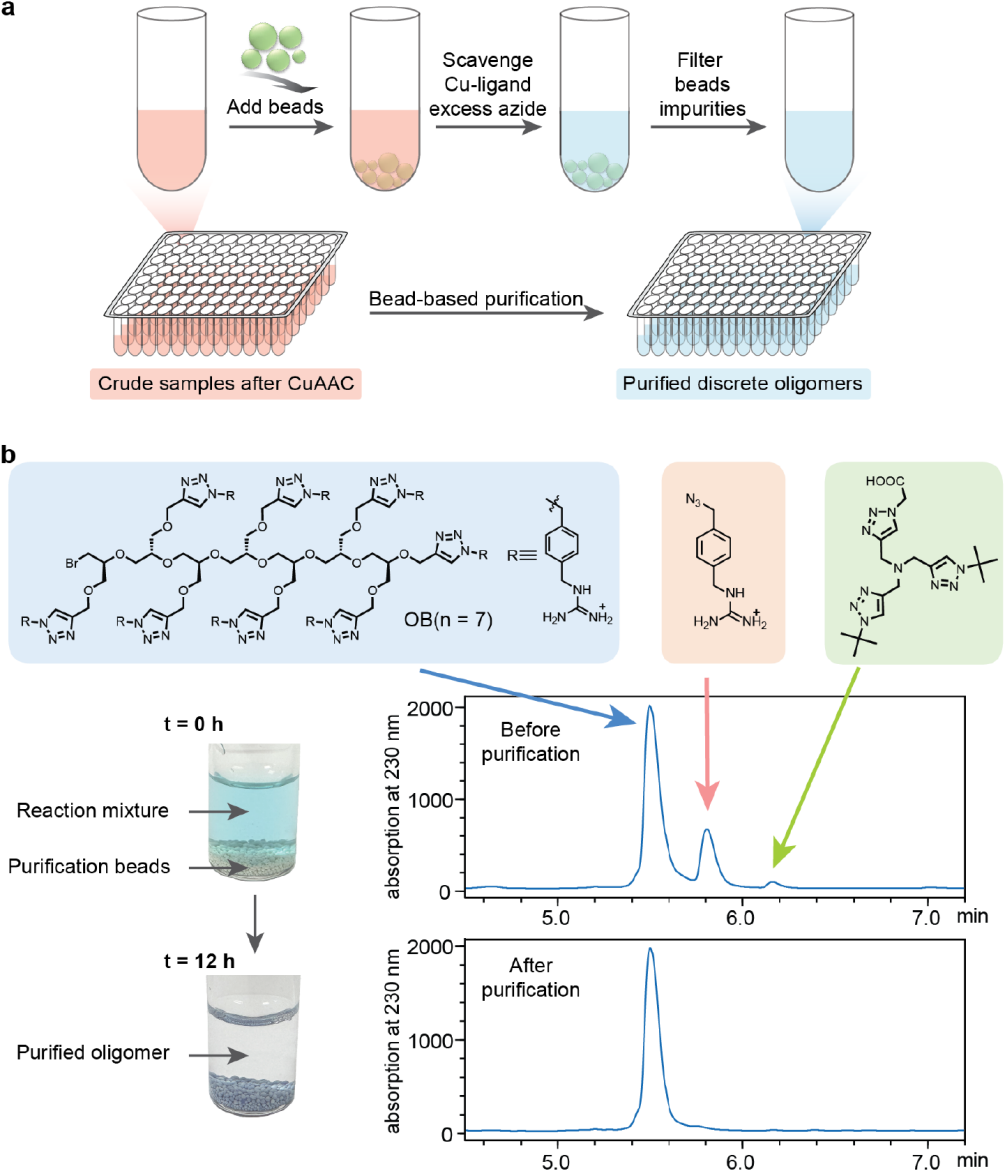
Bead-based purification. (a) Schematic illustration of high-throughput purification by using the beads to absorb and remove impurities from the oligomer solutions. (b) Color change and HPLC results for a representative oligomer before and after the beads purification.

The use of customized beads results in significant simplification of the synthesis and purification process for functionalized discrete oligomers over the entire library range (Figure 7a). Following click functionalization, the multifunctional beads are added to the aqueous reaction mixture to absorb copper ions, BTTAA ligands, and excess azido compounds at room temperature without stirring and then routinely filtered to afford the purified, discrete oligomers. As depicted in Figure 7b, after the click reaction between the heptamer (DP = 7) containing eight alkyne units and azido benzyl guanidinium, the reaction mixture exhibited a light blue color, indicating the presence of oxidized Cu^2+^. High performance liquid chromatography (HPLC) analysis revealed a prominent peak corresponding to the desired oligomer product as well as additional signals from the BTTAA ligand and excess azido benzyl guanidinium. After incubation at room temperature with the amine/DBCO functionalized beads, the solution becomes colorless while the beads are stained blue, qualitatively indicating that copper ions are absorbed into the beads and removed from the solution. Significantly, HPLC analysis of the purified heptamer of OB containing eight charged guanidinium units displays a single peak for the discrete oligomer with high mass recovery and no detectable peaks for BTTAA ligand and excess azido starting material. This contrast in sample purity, before and after bead purification, is mirrored by inductively coupled plasma (ICP) analysis, which revealed >99% copper removal (Figure S18), clearly demonstrating the efficacy of this purification step.

The success of this overall strategy, coupled with its simplicity, allows for the development of standardized reaction kits. Significantly, the reaction kits only require basic liquid transferring skills and further illustrate the power of combining click chemistry with multifunctional purification beads for accelerating the synthesis of discrete oligomer libraries (Figure S26). These kits contain alkyne oligomers, copper catalysts, azido compounds, and purification beads, respectively. This approach allows nonexperts to access discrete oligomer libraries based on controlled molar mass and different side chain structures by mixing certain reagents in a specific order. This ability to generate oligomer libraries offers significant opportunities to systematically investigate chain-length dependent properties. For illustrative purposes, these functionalized oligomer libraries were investigated as a versatile platform for understanding chain length and structure–activity relationships in the development of antibacterial agents.

### Discrete Oligomer Libraries for Antibacterial Performance

Functional synthetic oligomers and polymers have recently emerged as a promising class of materials to address the growing issue of antibiotic-resistant infections. These systems are designed to mimic natural antibacterial peptides and typically contain multiple cationic units with the polycationic structure targeting cell membranes and potentially nucleic acids.^46−49^ This mode of action makes them promising for treating antibiotic resistant infections.^50^ However, a significant obstacle is the higher toxicity of these polycationic macromolecules compared to traditional small molecule antibiotics, with a key challenge being to fully understand the role of different functional side chains and molar mass to balance efficacy and toxicity while increasing the therapeutic index. Due to the challenge of obtaining discrete macromolecules, current reports have primarily used disperse materials, which consist of a broad range of molar mass and structures. As a result, the biological behavior of each component is difficult to understand and the overall structure–function relationships are hard to establish. From both fundamental and applied viewpoints, it is therefore advantageous to use discrete materials for both research and clinical applications to identify the most active derivatives.

**Figure 8:**
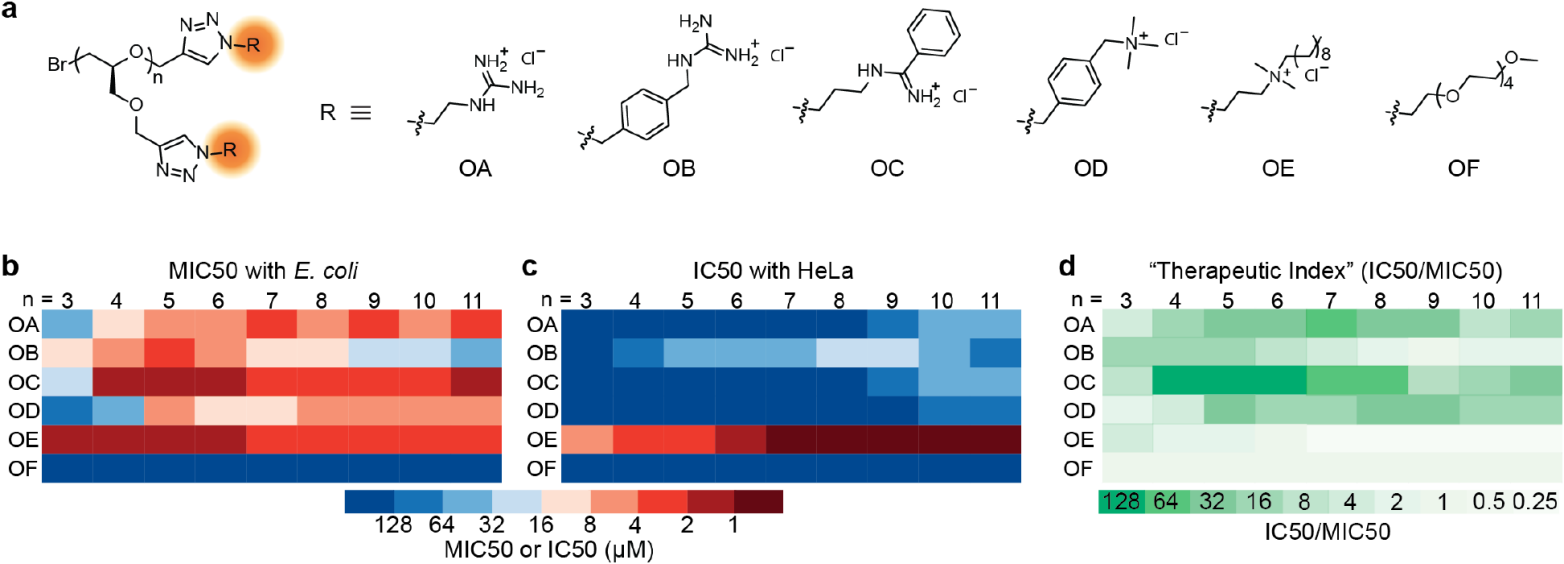
Antibacterial properties of the discrete oligomer library. (a) Chemical structures of the discrete oligomer library (OA, OB, OC, OD, OE and of represent different side chains). (b) Heat map of MIC50 values of the oligomers with **E. coli** cells. (c) Heat map of IC50 values of the oligomer library with HeLa cells. (d) Heat map of the apparent therapeutic index (IC50/MIC50) of the oligomers.

Using the high-throughput synthesis and purification platform described above, a library of 54 discrete oligomers based on (R)-glycidyl propargyl ether is reported. From an initial ring opening polymerization reaction, chromatographic separation gives nine oligomer lengths ranging from DP = 3 to DP = 11 (the number of alkyne units ranges from 4 to 12) and each of these derivatives is coupled with six different side chain functionalities to give the desired library of 54 discrete structures (Figure 8a). As a proof of concept, the antibacterial activities of this diverse range of oligomer samples are evaluated on *E. coli* (Figure 8b) and *B. subtilis* (Figure S30), representing Gram-negative and Gram-positive strains, respectively.^10^ Additionally, their cytotoxicity is assessed with HeLa cells representing mammalian cells. For all of the experiments, testing of the oligomers is based on molar concentration to better compare the performance of each derivative.

As shown in Figure 8b, the five cationic side chain oligomers exhibited antibacterial activities, with the minimum inhibitory concentration for 50% bacteria growth (MIC50) on *E. coli* cells ranging from 64 μM to less than 2 μM while the neutral penta(ethylene glycol) series displays high MIC50 values of greater than 128 μM for all oligomer lengths. Of particular interest is the observation that the relationship between chain length and efficacy is highly dependent on the nature of the different cationic oligomer groups. For example, discrete oligomers with aliphatic guanidinium side chains (OA) and benzyl trimethylammonium side chains (OD) show a decrease in MIC50 with increasing degrees of polymerization. In contrast, functionalization with benzyl guanidinium side chains (OB) leads to an initial increase in antibacterial activity from *n* = 3 to *n* = 5 followed by a decrease from *n* = 6 to *n* = 11. In contrast, phenyl amidinium side chains (OC) reveal a significant increase in efficacy from *n* = 3 to *n* = 4, with all oligomers from *n* = 4 to *n* = 11 exhibiting strong but similar potency against *E. coli*. Finally, the amphiphilic trimethylammonium functionalized oligomers (OE) provide antibacterial ability at all lengths tested, while no activity was observed for the PEG functionalized oligomers (OF).

The cytotoxicity of these oligomers on mammalian cells was then examined, with significantly different molar mass behavior being observed when compared to the MIC50 performance for *E. coli* described above. Again, both the nature of the side chain and the oligomer length are found to impact function. As illustrated in Figure 8c, the heat map depicts the half-maximal inhibitory concentration (IC50) of each oligomer for HeLa cells. Notably, the majority of oligomers showed lower toxicity with HeLa cells compared to either Gram-positive or Gram-negative bacteria, and the toxicity is observed to increase with DP. These results suggest that the toxicity of polydisperse antibacterial oligomers is predominantly attributable to the higher molar mass fractions. To illustrate this point, the apparent “therapeutic index” was calculated for each oligomer, by dividing the IC50 for HeLa cells by the MIC50 from *E. coli* (Figure 8d). Significantly, the oligomer with 8 aliphatic guanidinium groups (OA) and the oligomers with 5 to 7 phenyl amidinium groups show the highest indexes, implying the optimal performance for these specific oligomers when balancing efficacy and toxicity.

The concentration-dependent activity and toxicity of the discrete oligomer series based on aliphatic guanidinium side chains (OA) with *E. coli* and HeLa cells are illustrated in greater detail in Figure S28. Notably, the efficacy of the oligomer showed an average 100% increase with each repeat unit from DP = 3 to DP = 7. However, longer oligomer chains did not lead to increased activity, indicating that DP ≥ 7 is optimal for maximizing the antibacterial behavior. In terms of toxicity studies (Figure S28b), higher toxicity with increasing DP was observed under the concentrations tested, especially when DP > 8. Taken together, these results illustrate that DP = 7 represents the best balance between potency and toxicity for the oligomer series OA. These findings suggest that the correlation between chain length and biological activity is highly dependent on both side chain structure and oligomer length.

**Figure 9:**
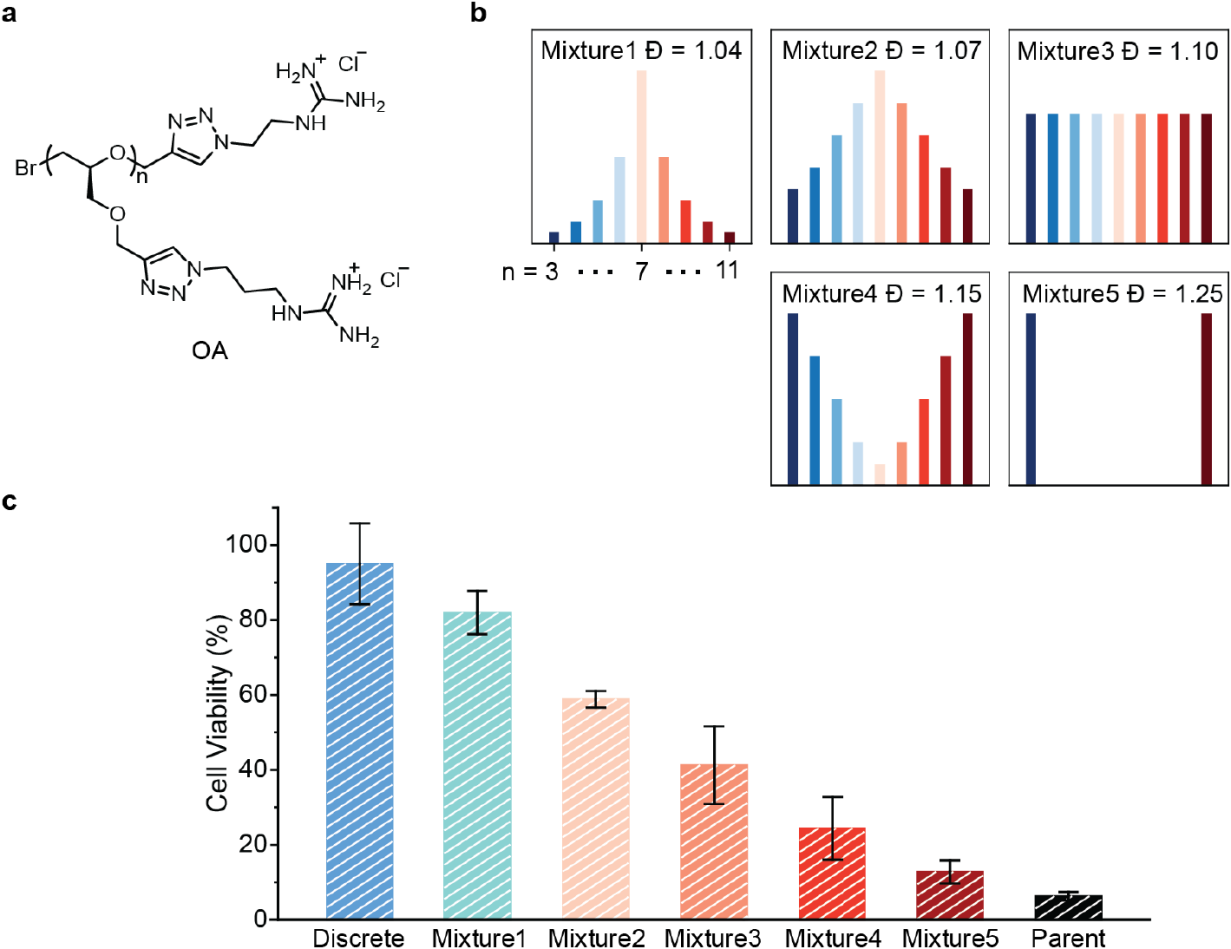
Dispersity-dependent behaviors. (a) The chemical structure of guanidinium containing oligomers (OA). (b) Schematic illustration of artificially formulated polydisperse oligomer mixtures with different molar ratios of each length. (c) Cell viability of HeLa cells treated with 128 μM oligomer OAs with different dispersities with DP = 7. Error bars represent ± SD of 3 replicates.

One of the advantages of preparing libraries of discrete oligomers is the ability to formulate mixtures with tailored dispersity.^51−53^ To investigate this novel possibility, discrete oligomers could be blended in different molar ratios to give the same average DP of 7 but with varying dispersity patterns (Figure 9a). The tailored mixtures could then be evaluated in comparison with both the discrete DP = 7 oligomer as well as the parent disperse sample before chromatographic separation, which has an average DP = 7, *Đ* = 1.15 (Figure S1). While the formulated mixtures demonstrate comparable antibacterial activities (Figure S35), there is a significant trend of decreasing HeLa cell viability with increasing dispersity (Figure 9b). Notably, the as-prepared parent sample is the most toxic, even though its dispersity index is lower than that of some tailored mixtures. This is likely due to the presence of high molar mass oligomers (DP > 11) in the parent samples which, based on the trends observed in this work, are significantly more toxic than lower molar mass oligomers. These findings suggest that disperse macromolecules with high molar mass components contain less active and, very importantly, more toxic fractions, leading to a reduced therapeutic index.

## Conclusion

This study presents a novel strategy for the synthesis and purification of discrete, highly functionalized oligomer libraries in a 96-well, high-throughput format. By combining living ring opening polymerization with chromatographic separation, a series of discrete alkyne oligomers based on (R)-glycidyl propargyl ether with 4 to 12 alkyne groups were isolated and fully characterized on multigram scale. The presence of multiple alkyne groups along the backbone allows post-functionalization via CuAAC click chemistry leading to significant chemical diversity. Key to the success of this high-throughput strategy is the development of multifunctional purification beads that absorb and react with impurities and excess reagents under mild and simplified conditions. This platform enables nonexperts to develop discrete oligomer libraries with specific DPs and varied side chain functionalities, providing opportunities for studying structure–activity relationships and chain-length dependent properties of functional oligomers. This is illustrated through the evaluation of discrete, cationic oligomers as antibacterial agents with the results highlighting the importance of a discrete structure, the number, and nature of the cationic side chains, as well as facile library preparation. This strategy provides a modular and scalable pathway to discrete synthetic oligomers that are relevant for a variety of biomedical and material applications.

## Supporting Information Available

General experimental procedures and detailed synthetic procedures and characterization and additional data (PDF)
