# Supplementary material for: High-Throughput Synthesis, Purification, and Application of Alkyne-Functionalized Discrete Oligomers
Source: J Am Chem Soc. 2024 Mar 15;146(12):8650–8. doi: 10.1021/jacs.4c00751 (PMC10979451; doi:10.1021/jacs.4c00751)
Supplement: Supplementary file 1 — ja4c00751_si_001.pdf [file ja4c00751_si_001.pdf]

High Throughput Synthesis, Purification, and Application of Alkyne Functionalized Discrete Oligomers

Junfeng Chen [†], Vittal Bhat[‡] and Craig J. Hawker*[,†]

[†]Materials Department, Materials Research Laboratory, and Department of Chemistry and Biochemistry, University of California, Santa Barbara, California 93106, United States.
[‡]Department of Chemistry, University of North Carolina, Chapel Hill, North Carolina 27599, United States;

Contents

Materials and Instruments ········· S2

Synthesis and Characterization ········· S3

Purification Bead Development ········· S19

High Throughput Post Functionalization and Purification ········· S21

Biological Assays and Results ········· S27

References ········· S34

Materials and Instruments:

All reagents were purchased from Sigma-Aldrich, Acros Organics, Fisher Scientific, TCI America, and AA Blocks, and used without further purification unless otherwise noted. For the synthetic procedures, toluene, THF, DCM, chloroform, and DMF were stored over activated 4 Å molecular sieves. NMR spectra were recorded using Bruker CB500 in the NMR facility, Department of Chemistry and Biochemistry, University of California, Santa Barbara. Mass spec and HPLC experiments were performed using Bruker Microflex LRF MALDI-TOF and Shimadzu Nexera Hybrid HPLC at the BioPACIFIC MIP, University of California, Santa Barbara. Circular dischroism (CD) spectra and UV spectra were collected using Jasco J-1500 CD Spectrapolarimeter at the Optical Characterization Facility, University of California, Santa Barbara. Biological experiments were performed using the Biocore facility at California Nanosystem, University of California, Santa Barbara. NMR spectra were processed by using MestReNova (v14.0). All the other RAW data files were processed using OriginPro2022 and imported into Adobe Illustrator CC 2022 or Microsoft PowerPoint for coloring and annotation.

MALDI Mass Spectrum Measurements

The MALDI experiments were performed by using dithranol as the Matrix and NaTFA as the ion source. The measurements were conducted on a Bruker Microflex LRF MALDI TOF instrument with the RP-peptide method.

SEC Spectrum Measurements

The SEC experiments were performed on an SEC using chloroform as the elution solvent.
Pump: Waters Alliance HPLC System, 2690 Separation Module.
Two columns: Agilent, PLgel, 5 µm MiniMIX-D, 250 x 4.6 mm + guard (MW linear range 200 - 400,000 g/mol)
Detectors:
Waters 2410 Differential Refractometer (RI)
Waters 2998 Photodiode Array Detector (PDA)
Solvent: Chloroform with 0.25% (v/v) TEA
Flow Rate: 0.35 mL/min
Injection: 40 µL.

Synthesis and Characterization

Preparation of the polydisperse oligomer

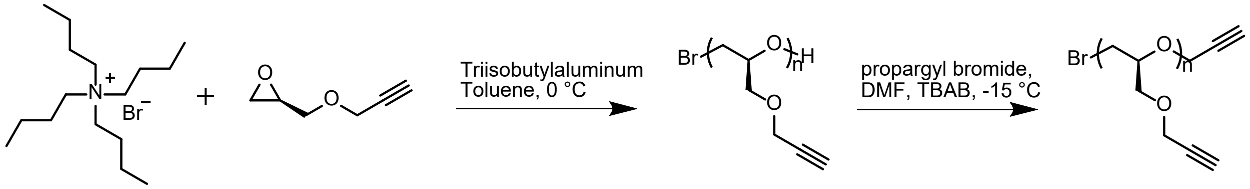

In a 200 mL round bottom flask, R-propargyl glycidyl ether (11.5 g, 102 mmol) and TBAB (6.31 g, 19.6 mmol) were dissolved/suspended in 50 mL of dry toluene. The mixture was cooled in an ice bath under argon. To the mixture was added triisobutylaluminium (22.4 mL, 1 M in toluene), and the mixture was stirred in an ice bath for 1 h, and the ice bath was removed and the mixture was stirred at r.t. for another 2 h. To the mixture was added 2 mL of EtOH to quench the reaction, and stirred for 5 min at r.t.. The mixture was diluted with 100 mL of chloroform and washed twice with 100 mL of 0.1 M HCl, 100 mL of water, and 20 mL of brine. The organic layer was dried over Na_2SO_4, filtered and concentrated by using a rotary evaporator. Without further purification, the crude product was dissolved in 20 mL of DMF, and propargyl bromide (5.54 g, 37.2 mmol) and TBAB (0.5 g, catalytic amount) were added to the DMF solution. The mixture was cooled in a brine-ice bath at around -15 ˚C, and NaH (1.48 g, 37.2 mmol) was added carefully. The mixture was vigorously stirred for 30 min and the reaction mixture was quenched by adding 20 mL of saturated NH_4Cl(aq). The mixture was diluted with 200 mL of water and extracted twice with 100 mL of DCM. The combined DCM solution was washed twice with 100 mL of water and 20 mL of brine. The organic layer was dried over Na_2SO_4, filtered, and concentrated by using a rotary evaporator. The crude mixture was characterized by nuclear magnetic resonance (NMR), size exclusion chromatography (SEC), and matrix-assisted laser desorption/ionization (MALDI).

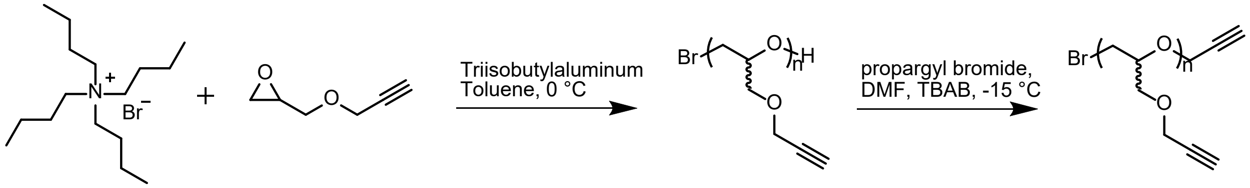

The synthesis of the atactic oligomer followed the same procedure as described above, except that an equal (1:1) mixture of R and S isomer of glycidyl propargyl ether was used as the monomer during the polymerization.

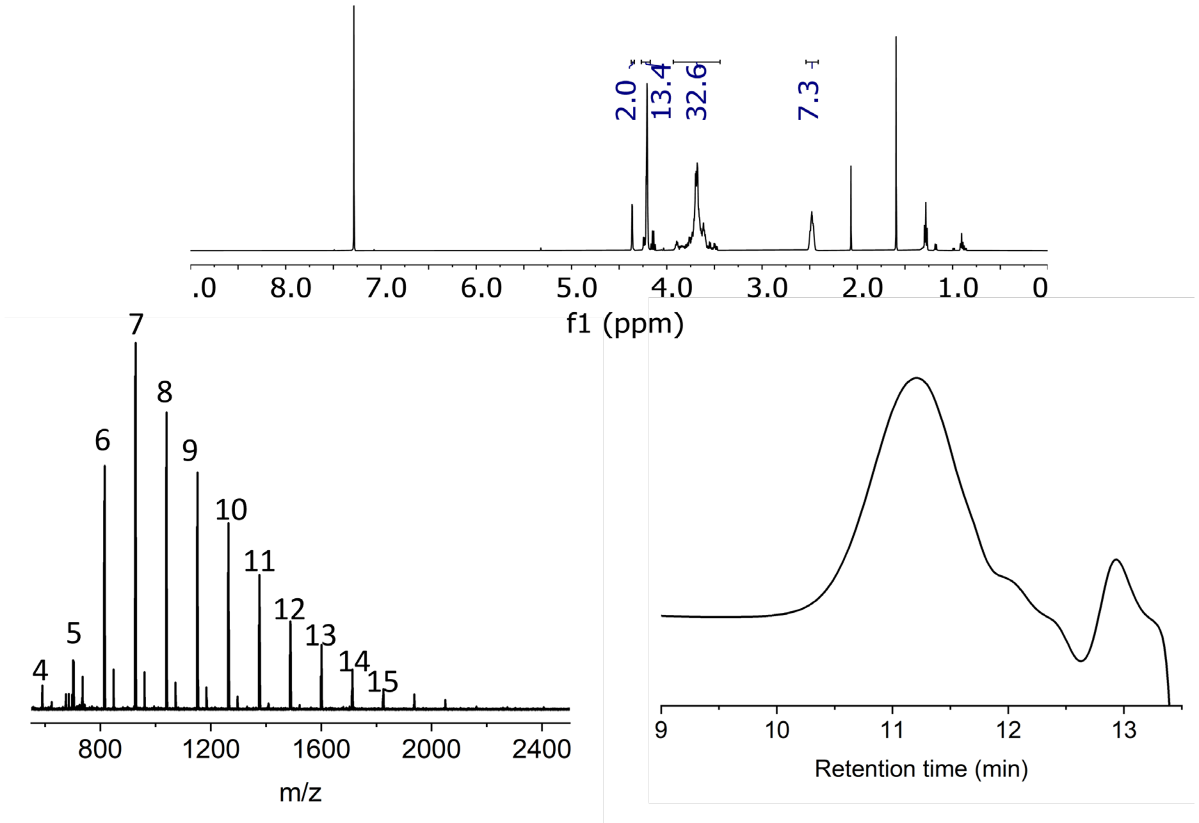

Figure S1. 1H NMR, MALDI, and SEC spectra of the crude disperse oligomer.

Automated Chromatography Separation

The chromatography was performed on a Biotage automatic chromatograph system. Generally, the Biotage® Sfär Silica column (350 g) was loaded onto the Biotage automated chromatography instrument and rinsed with hexane. To the column was added 12 g of the crude mixture of the oligomer in 10 mL of DCM, and the chromatography separation was started with flow rate = 200 mL/min, using an evaporative light-scattering detector (ELSD) as the signal detector. The chromatography used a solvent gradient from 100% hexane to 50% (v/v) ethyl acetate in hexane. The fractions were automatically collected into 25 mm glass test tubes (50 mL each), and the MALDI spectra were tested to combine pure discrete oligomer fractions. The combined solution was dried using a rotary evaporator to afford colorless oils.

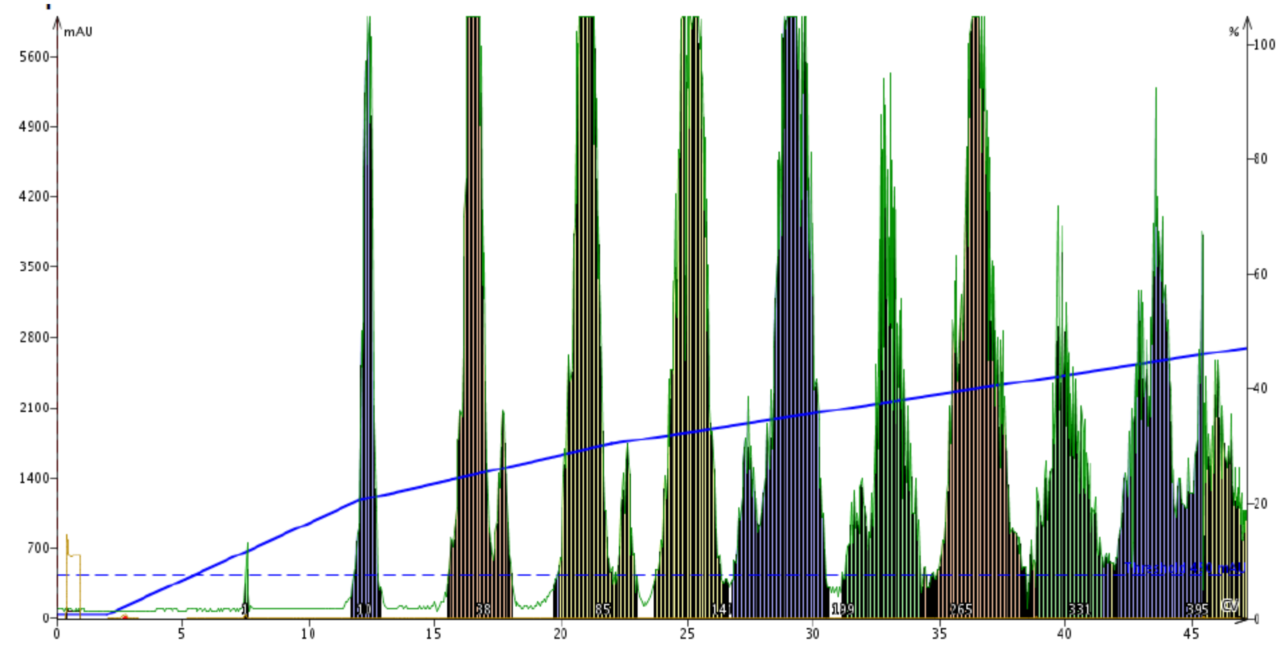

Figure S2. ELSD signalss of the chomatography separation.

NMR Spectra of oligomer samples.

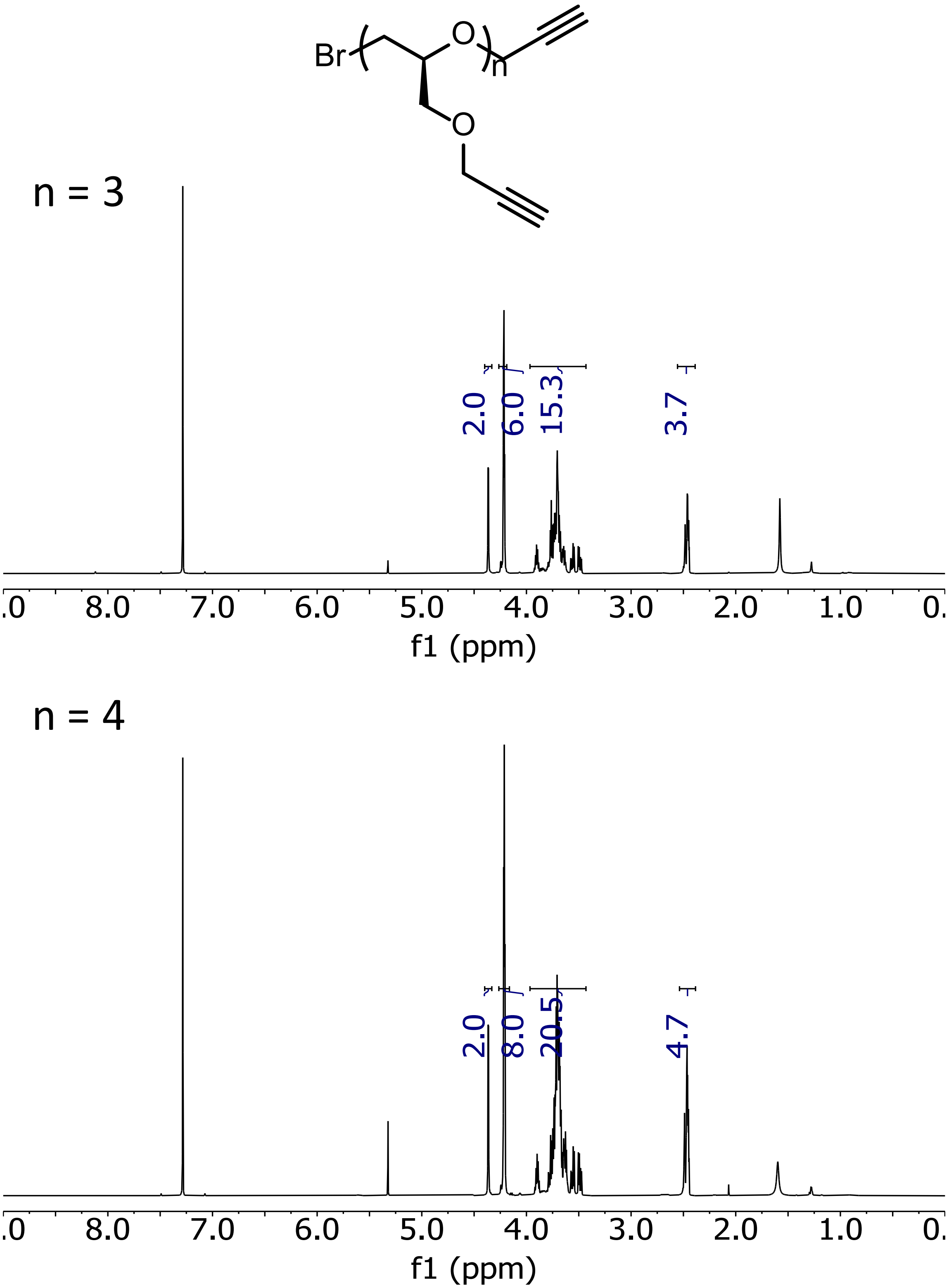

Figure S3. ¹HNMR spectra of the discrete oligomers (n = 3 and n = 4).

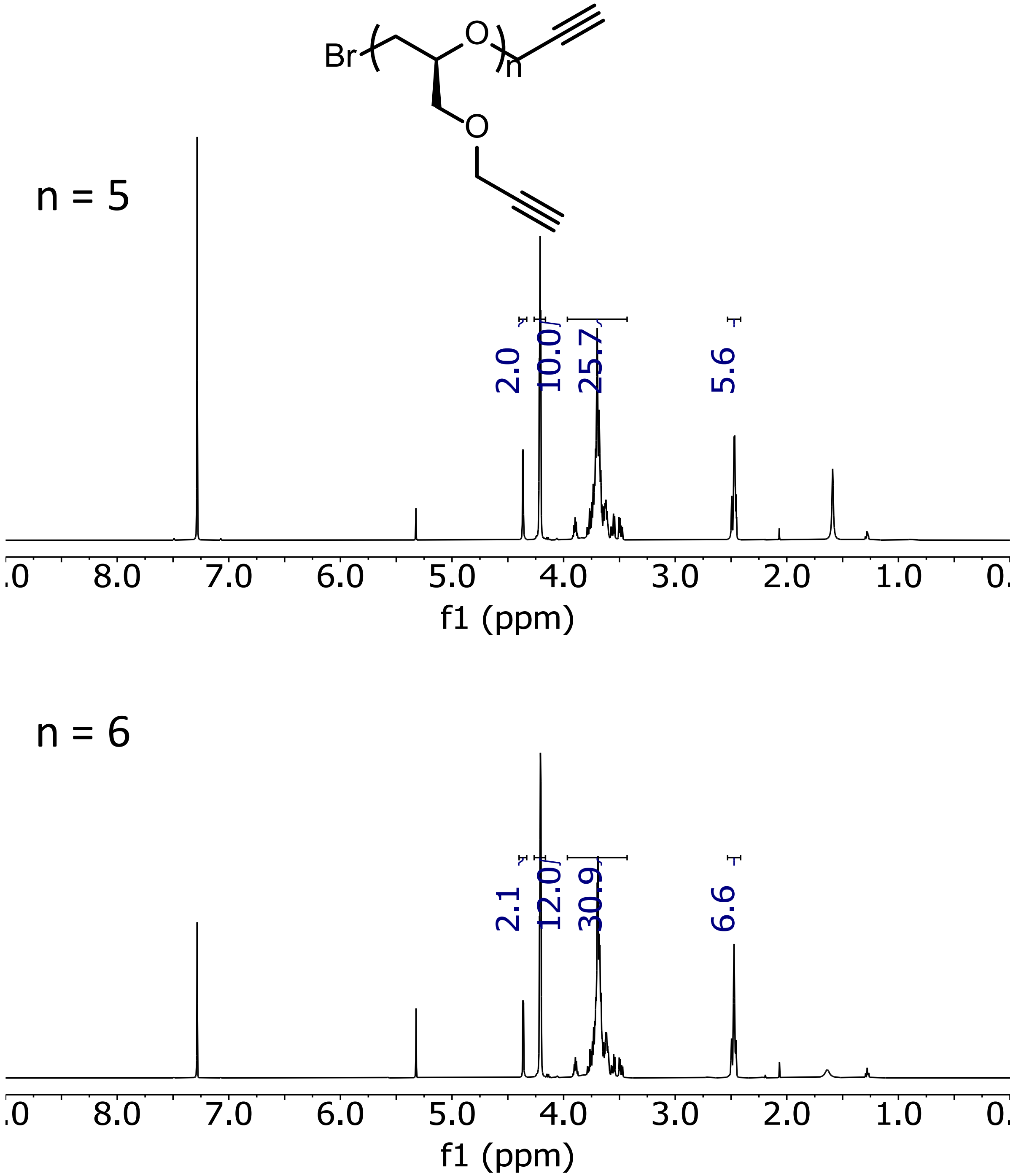

Figure S4. ^{1}HNMR spectra of the discrete oligomers (n = 5 and n = 6).

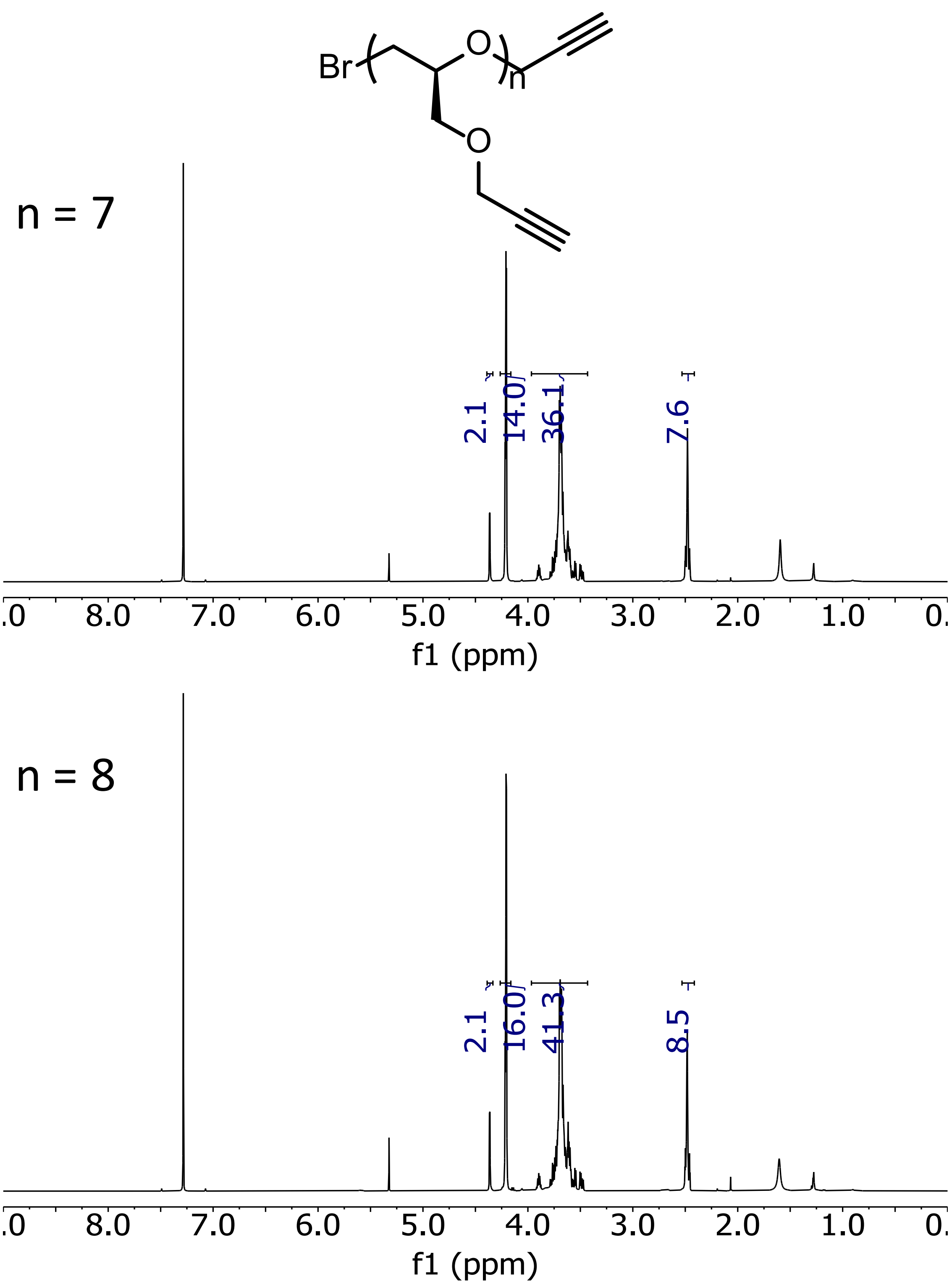

Figure S5. ^{1}HNMR spectra of the discrete oligomers (n = 7 and n = 8).

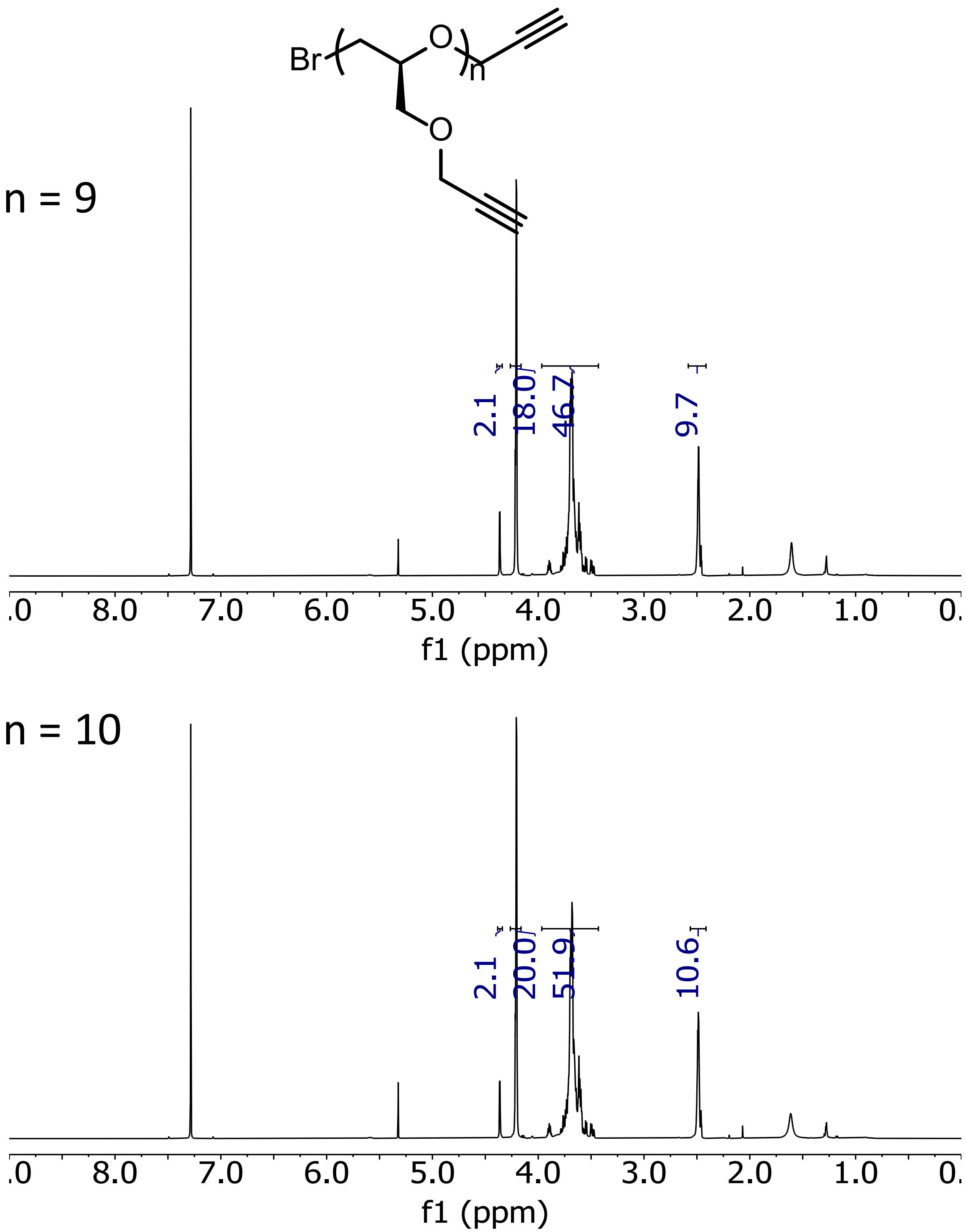

Figure S6. ^{1}HNMR spectra of the discrete oligomers (n = 9 and n = 10).

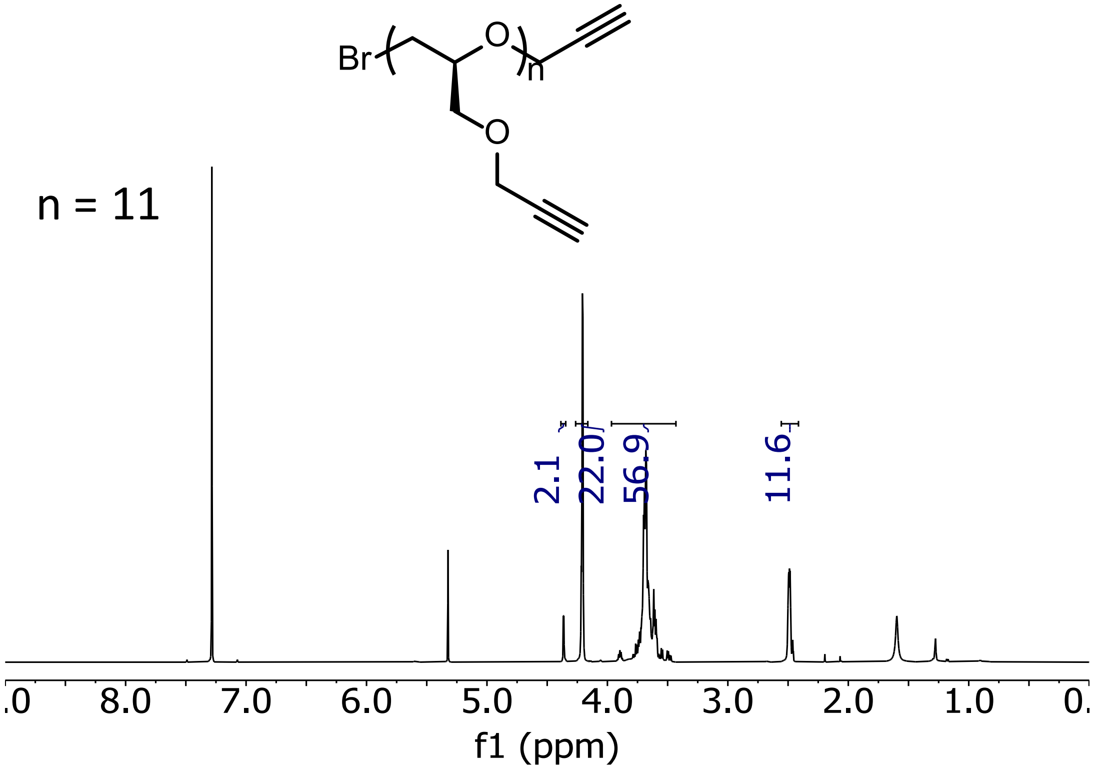

Figure S7. ^{1}HNMR spectra of the discrete oligomers (n = 11).

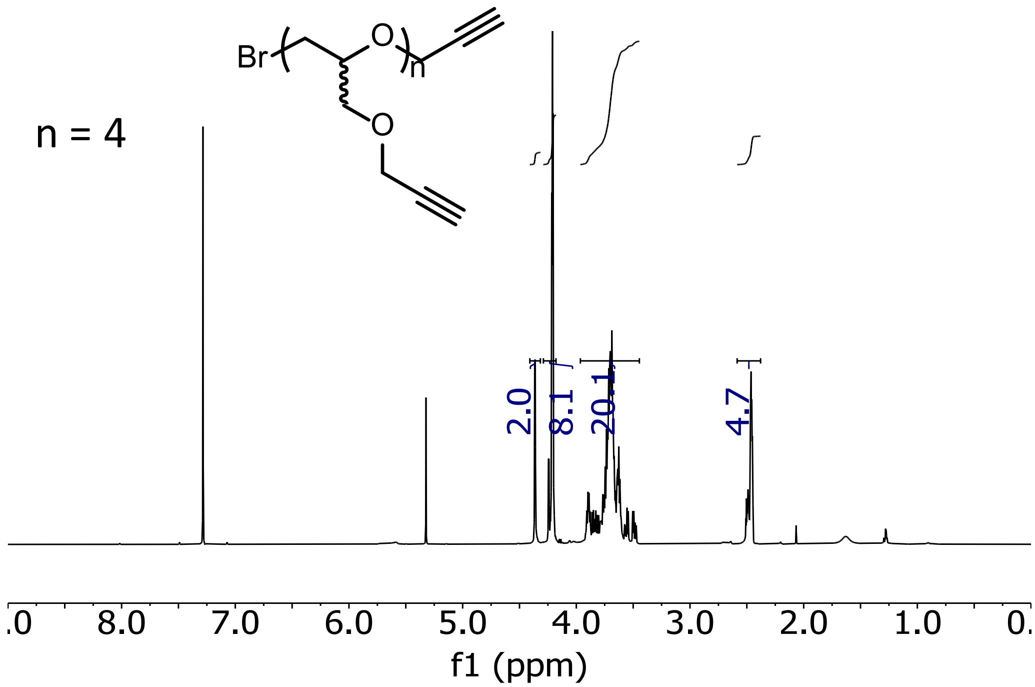

Figure S8. ^{1}HNMR spectra of the single molar mass atactic oligomers (n = 4).

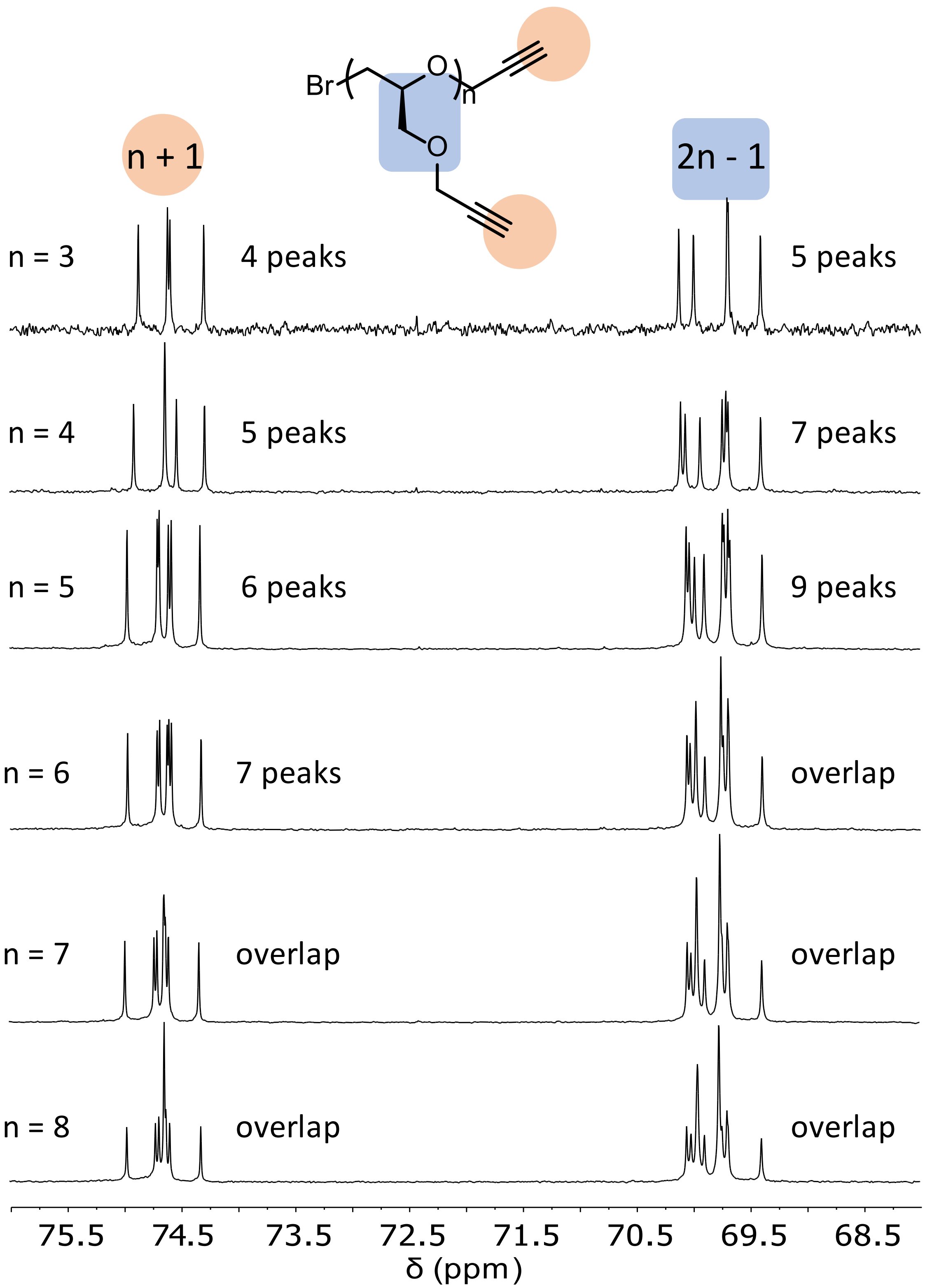

Figure S9. ^{13}CNMR spectra of the discrete oligomers from n = 3 to n = 8. Continued on the next page

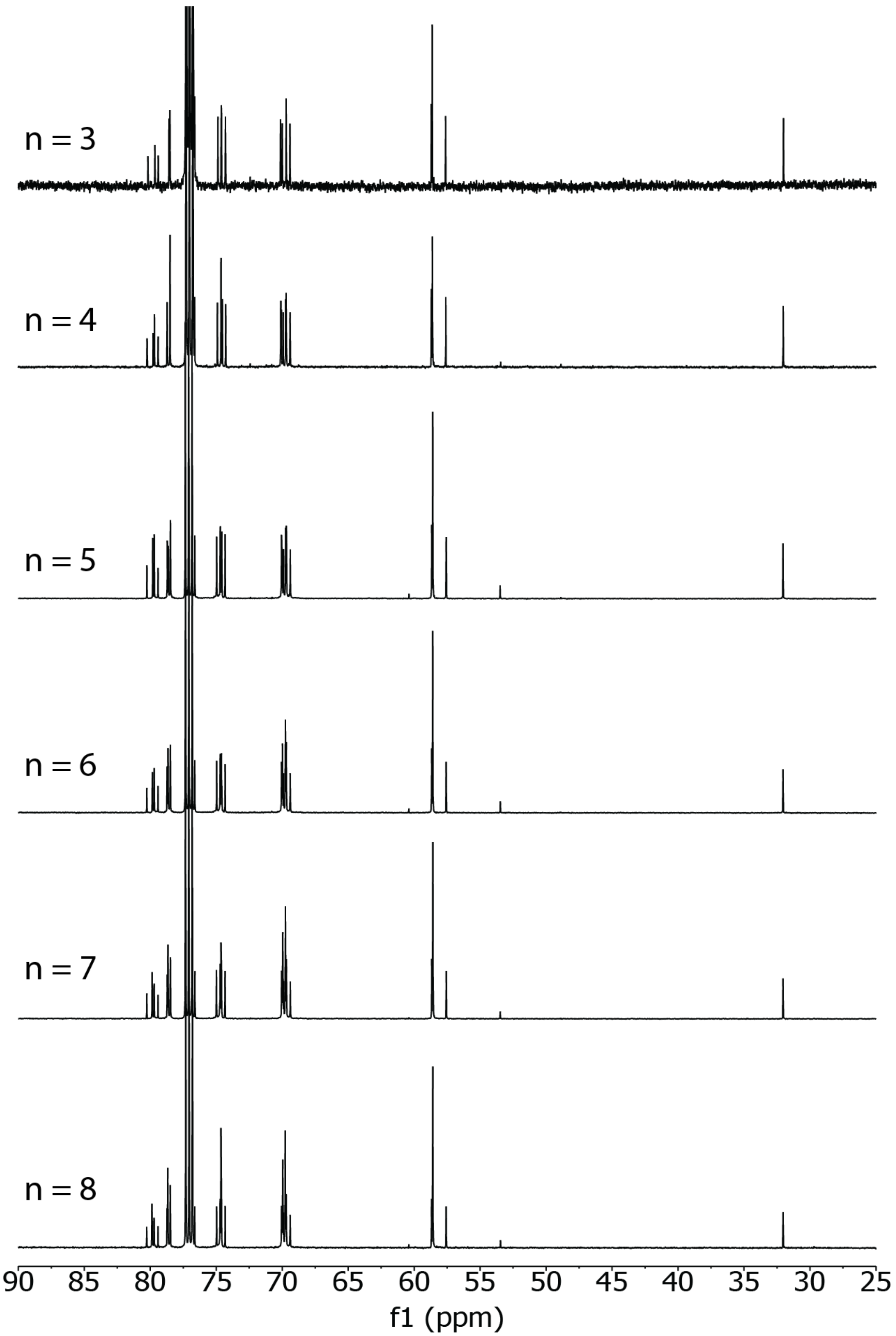

Figure S9. ^{13}CNMR spectra of the discrete oligomers from n = 3 to n = 8.

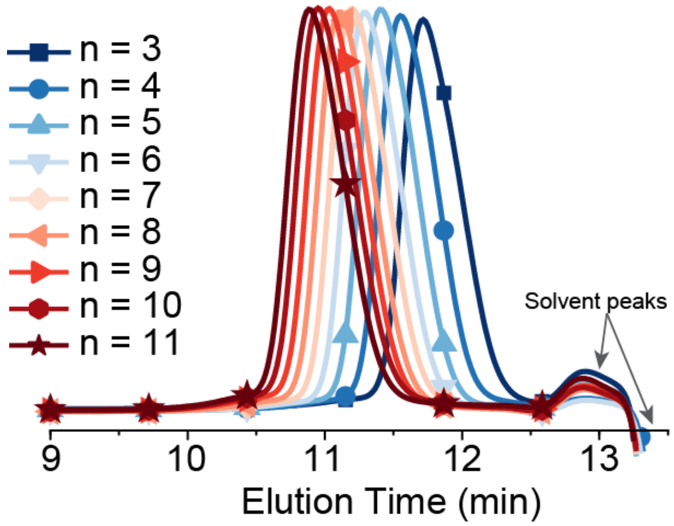

Figure S10. SEC spectra of the discrete alkyne containing oligomers.

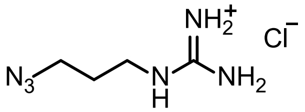

Azide**1**

Azide**1** was prepared by using a protocol reported in the literature.[1]

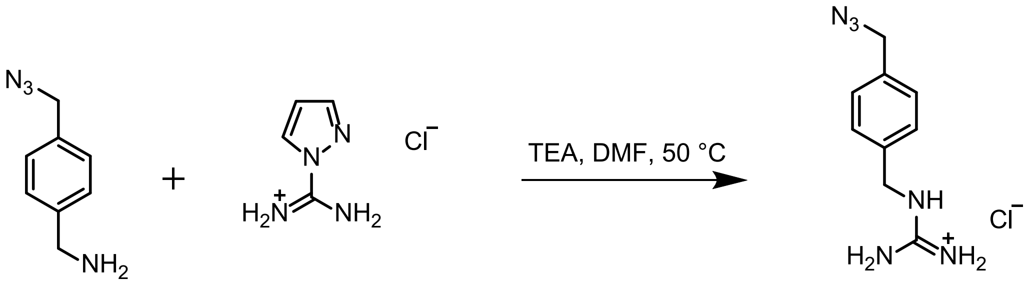

1 **2** Azide**2**

In a 20 mL glass vial, 1.5 g (9.3 mmol) of **1**, 1.4 g (9.3 mmol) of **2**, and 1.4 g (14 mmol) of triethylamine were dissolved in 5 mL of DMF, and the mixture was stirred at 50 °C for 24 h. The mixture was cooled to r.t. and precipitated in 40 mL of ether. The resulting viscous solid was washed 40 mL of ether 5 times and recrystallized in ethanol to afford 2.0 g (90%) of a white powder as Azide**2**.

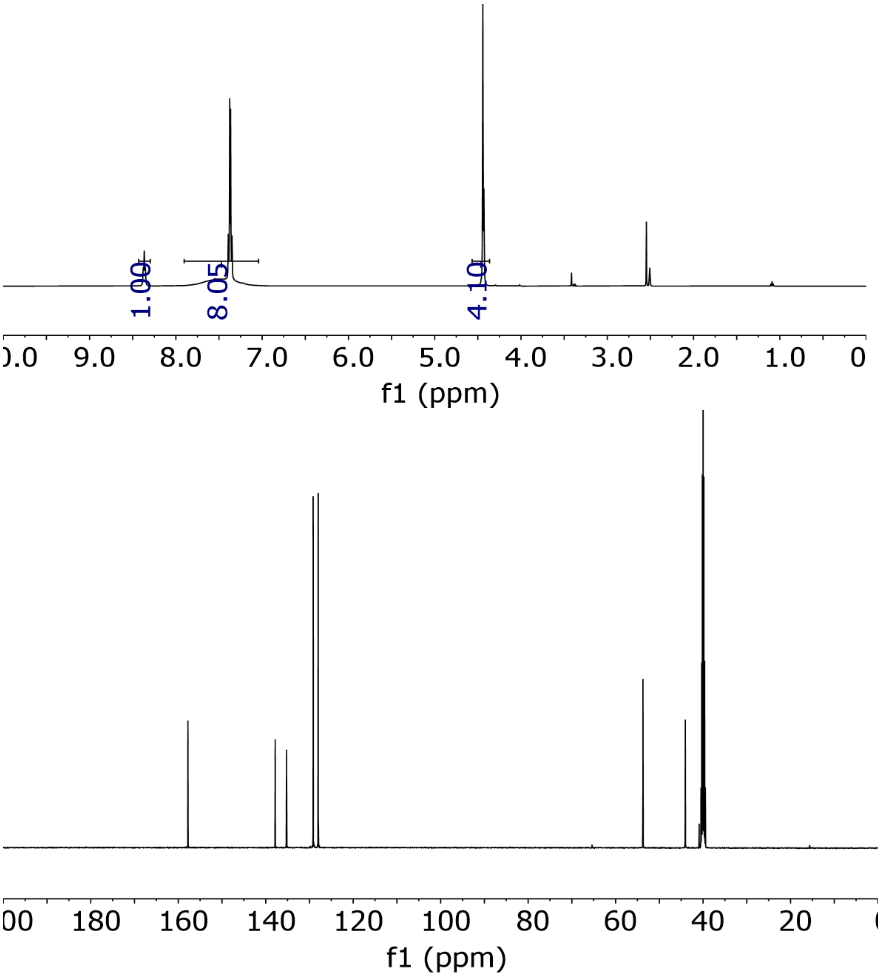

Figure S11. ^{1}HNMR and ^{13}CNMR spectra of Azide**2**.

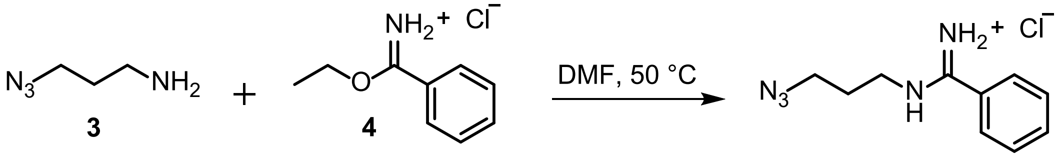

Azide**3**

In a 20 mL glass vial, 1.2 g (12 mmol) of **3** and 1.9 g (10 mmol) of **4** were dissolved and stirred in 5 mL of DMF at 50 °C for 12 h. The mixture was cooled to r.t. and precipitated in 40 mL of ether. The resulting viscous solid was washed 40 mL of ether 5 times and recrystallized in ethanol to afford 1.8 g (75%) of a white powder as Azide**3**.

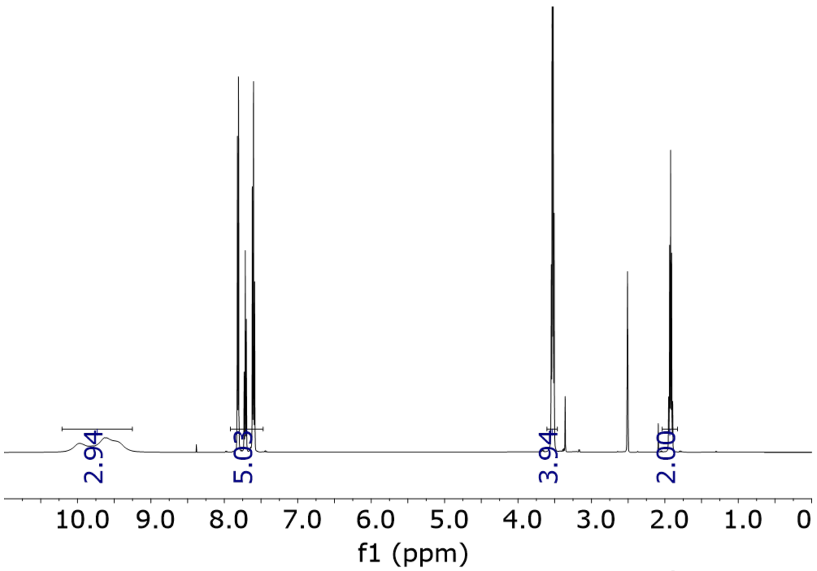

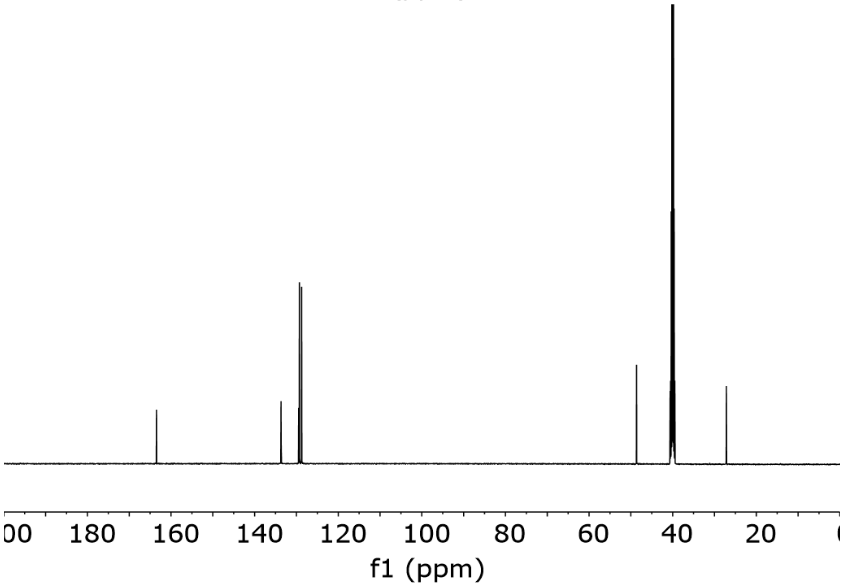

Figure S12. ^{1}HNMR and ^{13}CNMR spectra of Azide**3**.

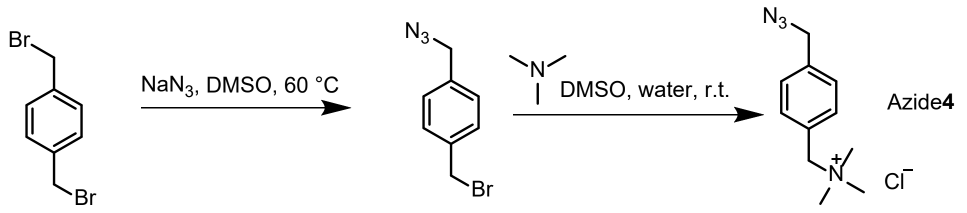

In a 40 mL glass vial, 5.0 g (19 mmol) of 1,4-Bis(bromomethyl)benzene and 1.2g (19 mmol) of sodium azide were stirred in 15 mL of DMSO at 50 °C for 6 h. The mixture was cooled in an ice bath, and 5.2 g (38 mmol) of trimethlyamine(43 wt%, aq) was added dropwise. The mixture was stirred at r.t. for 12 h. The solution was diluted with 200 mL of water and extracted twice with 100 mL of a 10:1 (v/v) mixture of chloroform and IPA. The combined organic layer was washed with 50 mL of water, and then 50 mL of brine 3 times, dried over $CaCl_2$, filtered, and concentrated by using a rotary evaporator. The crude product was purified by using silica chromatography with a solvent gradient from DCM to 20% (v/v) EtOH in DCM to afford 1.5 g (33%) of a white solid as Azide**4**.

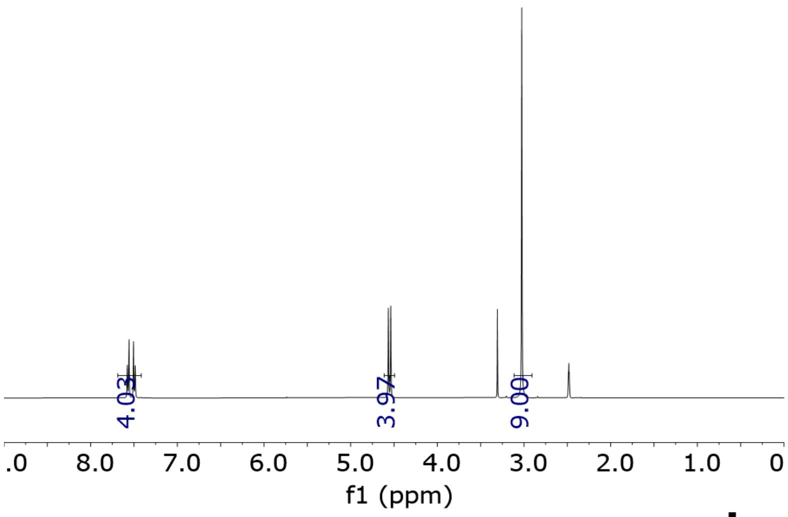

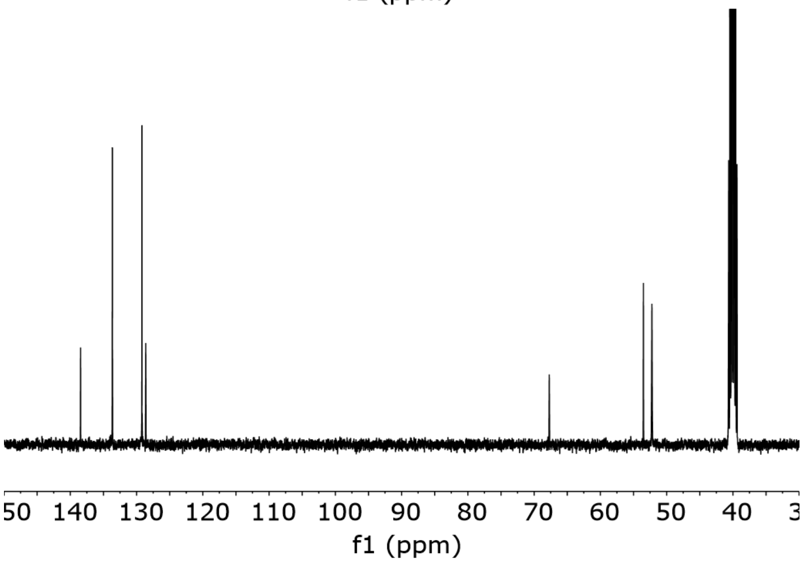

Figure S13. ^{1}HNMR and ^{13}CNMR spectra of Azide**4**.

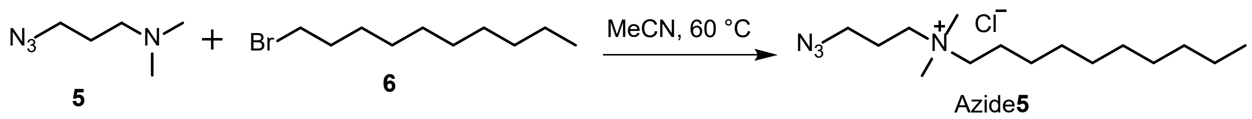

In a 20 mL glass vial, 1.3 g (10 mmol) of **5** and 4.4 g (20 mmol) of **6** were dissolved in 10 mL of MeCN and stirred at 60 °C for 12 h. The mixture was added to 100 mL of brine and extracted with 100 mL of DCM. The organic layer was washed with 50 mL of brine three times, dried over $CaCl_2$, filtered, and concentrated by using a rotary evaporator. The crude product was purified by using silica chromatography with a solvent gradient from DCM to 20% (v/v) EtOH in DCM to afford 1.2 g (39%) of a colorless liquid as Azide**5**.

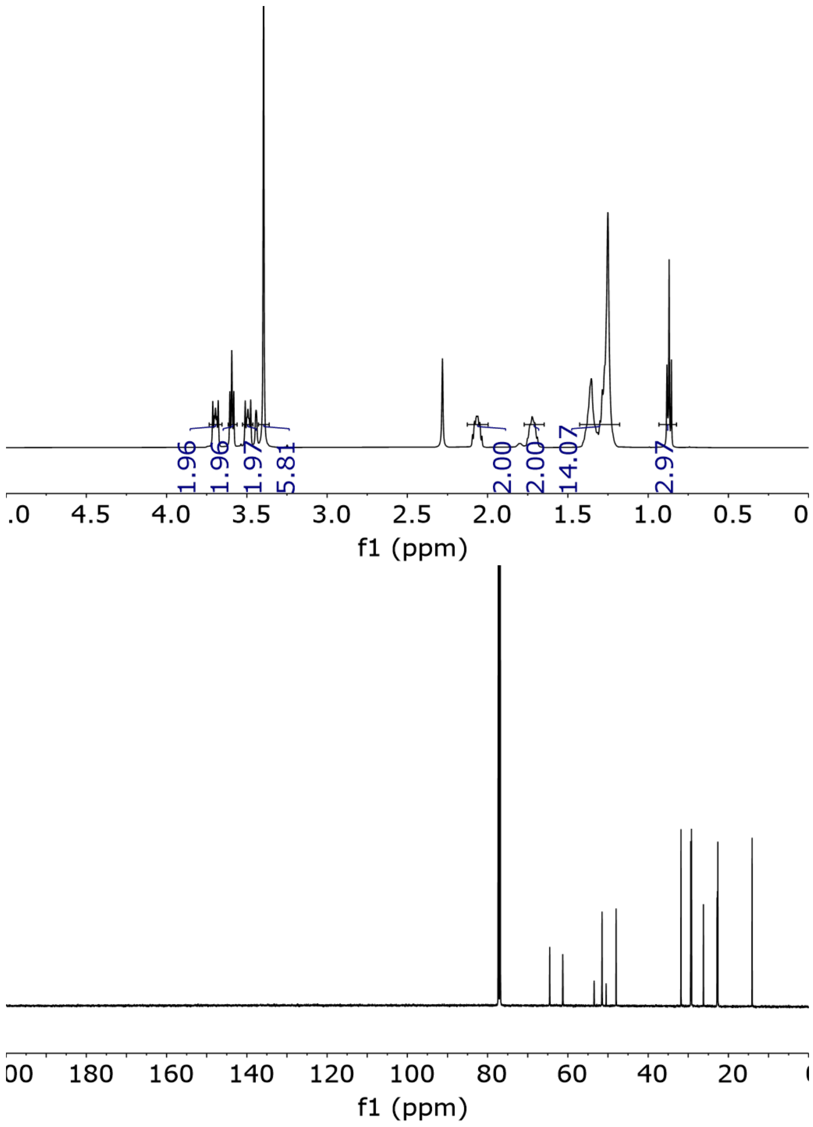

Figure S14. ^{1}HNMR and ^{13}CNMR spectra of Azide**5**.

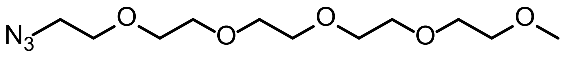

Azide**6**

Azide**6** was prepared by using a protocol reported in the literature.[2]

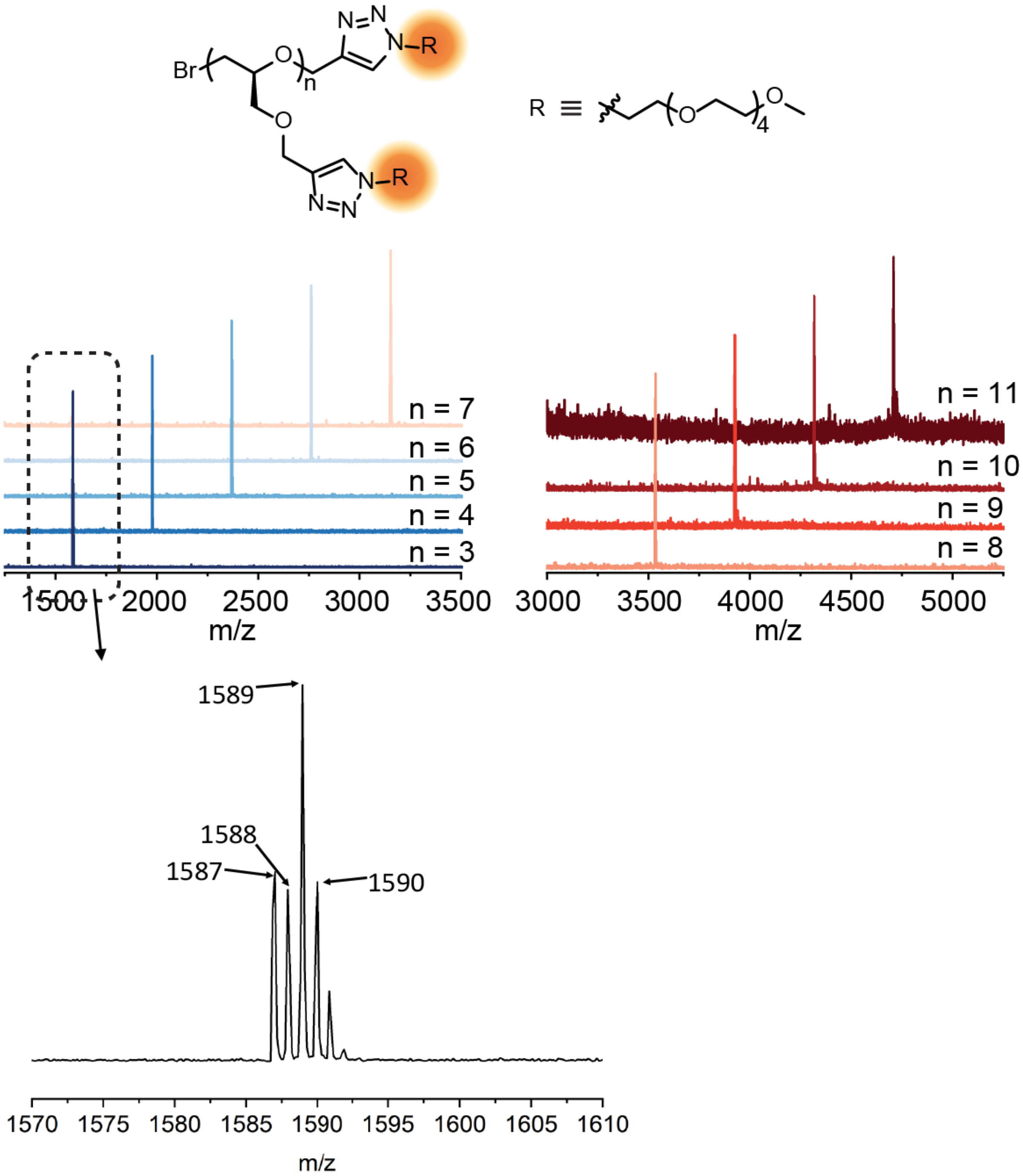

Figure S15. MALDI spectra of PEG functionalized discrete oligomers.

Purification Bead Preparation

The purification beads were developed based on the Diaion™ WA21J ion exchange beads obtained from Fishersci, which is a type of polystyrene beads containing diethylenetriamine ligands and divinylbenzene as the crosslinkers.

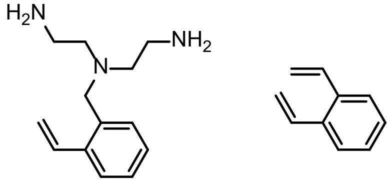

The purification beads were developed in 3 steps.

Water removeal from beads.

To a filtration funnel, 10 g of Diaion WA21J ion exchange beads was added and washed with 50 mL of ethanol 5 times to exchange the water content into ethanol. Then the beads were washed with 50 mL of chloroform 5 times to exchange the ethanol residue into chloroform. Then the beads were dried under a high vacuum at 50 °C for 24 hours to remove solvents.

Beads modification with DBCO.

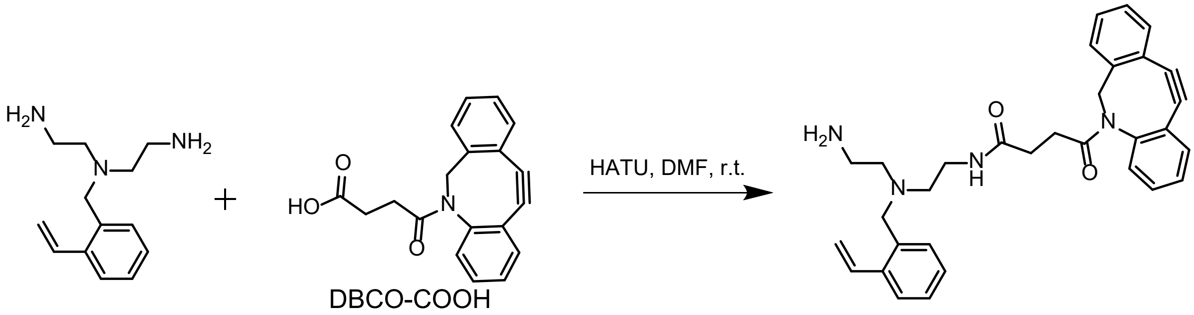

In a 100 mL round bottom flask, 0.61 g (2.0 mmol, ~10 mol% to the 1,2-ethanediamine) of DBCO-COOH was dissolved in 30 mL of molecular sieve dried DMF.[3] The DBCO solution was added 1.1 g of HATU (3.0 mmol, 1.5 eq to the DBCO) and 0.30 g of TEA (3.0 mmol, 1.5 eq to the DBCO), and the mixture was stirred at r.t. for 5 min. Then, 10 g of dried Diaion WA21J ion exchange beads were added to the mixture, and the reaction was left to proceed for 24 h.

Due to the challenge of characterizing crosslink polymer beads, the modification was validated by checking the residue DBCO compound in the amide coupling solution. Small aliquots of the solution during the HATU coupling reaction were taken and added to a DMF solution containing an excess amount of butylamine, and the residue amount of DBCO in the solution was measured by using a HPLC. As shown in Figure S12, the DBCO peak dispersed after 24 h of the amide coupling reaction, indicating that the DBCO group has been conjugated with the polymer beads in high conversion.

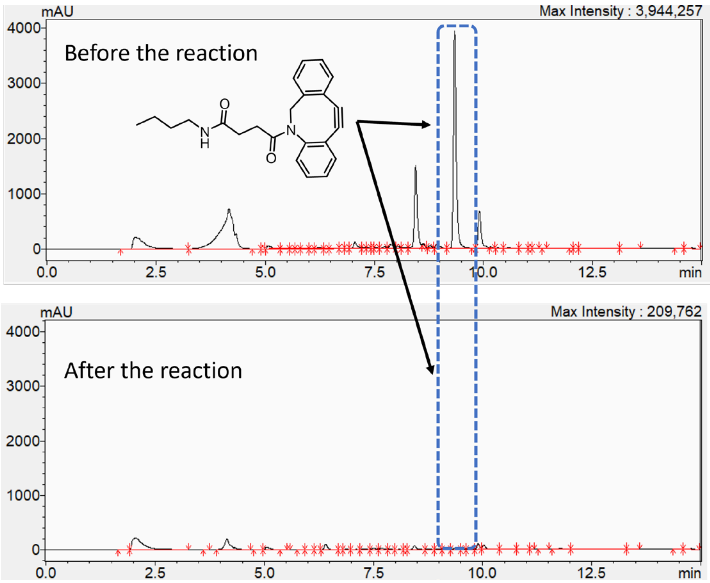

Figure S16. The HPLC trace of the DBCO compound in the solution before and after the bead modification.

Beads clean up.

After the HATU coupling reaction, the purification beads were washed extensively to remove any unbound residue chemicals. The beads were transferred to a filtration funnel, and they were washed with 50 mL of DMF 5 times, 50 mL of 0.1 M HCl in ethanol 5 times, and 50 mL of water 5 times. Then the beads were dried under a high vacuum to remove water content to be ready for use in purification. The fourier transform infrared (FTIR) spectra of the beads were obtained by using a Thermo Nicolet iS10 FTIR Spectrometer.

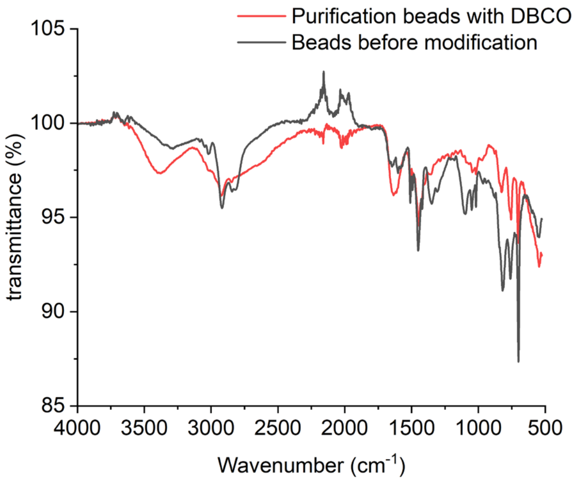

Figure S17. FTIR spectra of the purification beads before and after modification with DBCO groups.

High Throughput Post Functionalization and Purification

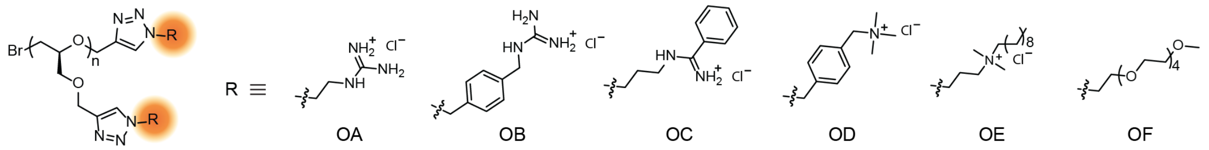

The stock solutions were prepared as follows:
Each discrete oligomer was dissolved in ethanol at a concentration of 20 mM.
Each azido compound was dissolved in water at a concentration of 100 mM.
BTTAA and $CuSO_4$ were mixed and dissolved in water at a concentration of 100 mM for both of them.
Sodium ascorbate was dissolved in water at a concentration of 200 mM.

In a deep 96-well plate (1 mL volume for each well), to each well was added 180 μL of discrete oligomer solution and 1.1 molar eq of azido compound compared to the alkyne concentration (160 to 480 μL from n = 3 to n = 11). The total volume of each well was normalized to 700 μL by adding water. To each well was added 7 μL of BTTAA-Cu solution and 10 μL of sodium ascorbate solution under argon in a glove bag. The click reactions were allowed to proceed in the 96-well plate at 50 °C for 3 h without stirring, and the mixtures were cooled to r.t. after the reaction. To each well was added 100-150 mg of purification beads, and the mixtures were incubated at r.t. without stirring for 12 h to absorb the impurities onto the beads. The purification beads were removed by using a 96-well plate filter, and the beads were washed twice with 500 μL of water to achieve high sample recovery. The washing aqueous solutions were combined with the oligomer solutions, and they were diluted with PBS buffer with the concentration normalized to 1 mM for the oligomer.

Inductively Coupled Plasma (ICP) Analysis

The ICP analysis was performed on one oligomer sample (OF(n = 7), entry 1) before the bead purification and two oligomer samples (OF(n = 7), entry 2; OA(n = 7), entry 3) after the bead purification. The ICP measurements were conducted on a Thermo ICAP 6300 model with 100 times dilution on the original solutions (5 mM of the oligomer). The amount of residue copper was measured and multiplied by 100 to reflect the copper content in the original solutions (Figure S11). The ICP results indicate that after the bead purification, 99% of the copper content has been removed.

Entry	Copper content
1	78 ppm
2	1 ppm
3	Undetected

Figure S18. The ICP analysis of oligomer samples before and after the bead purification .

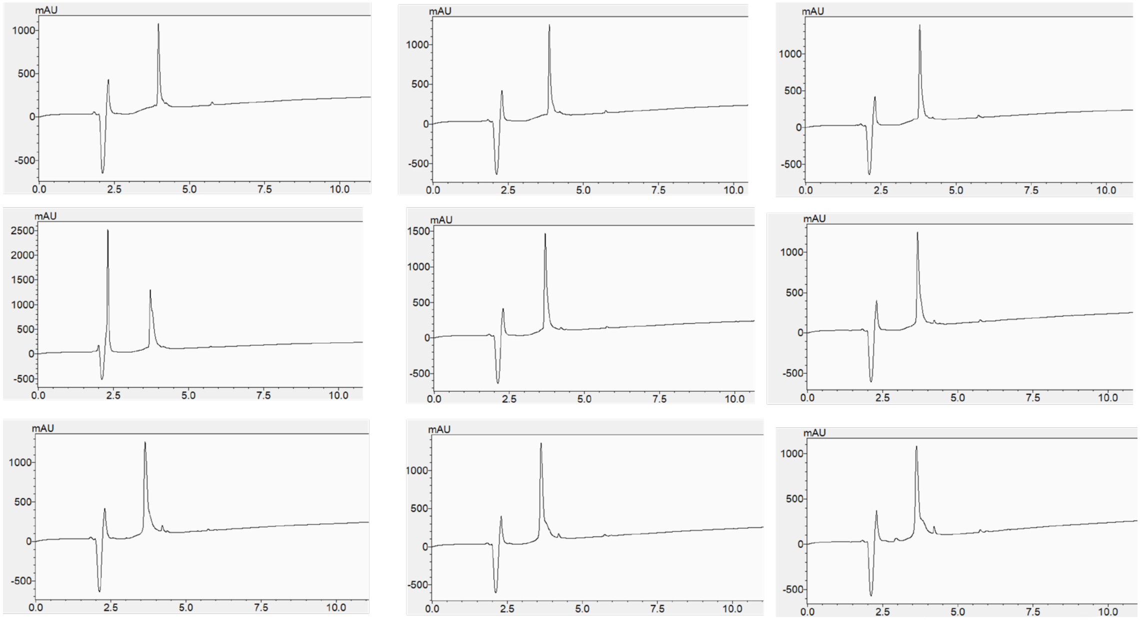

Figure S19. The HPLC results of oligomer OA samples after the bead purification, from n = 3 to n = 11.

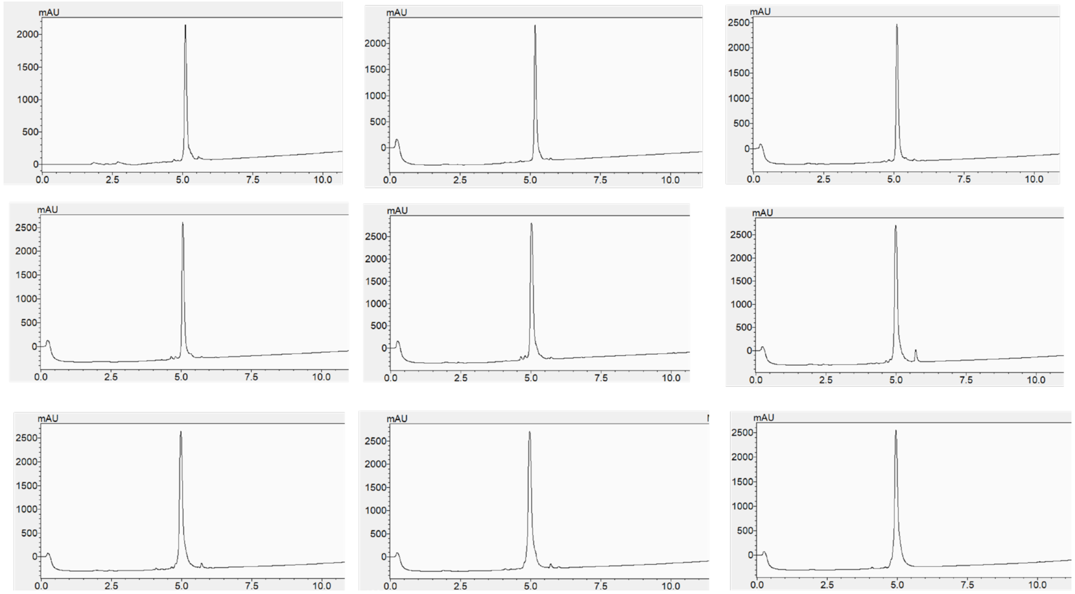

Figure S20. The HPLC results of oligomer OB samples after the bead purification, from n = 3 to n = 11.

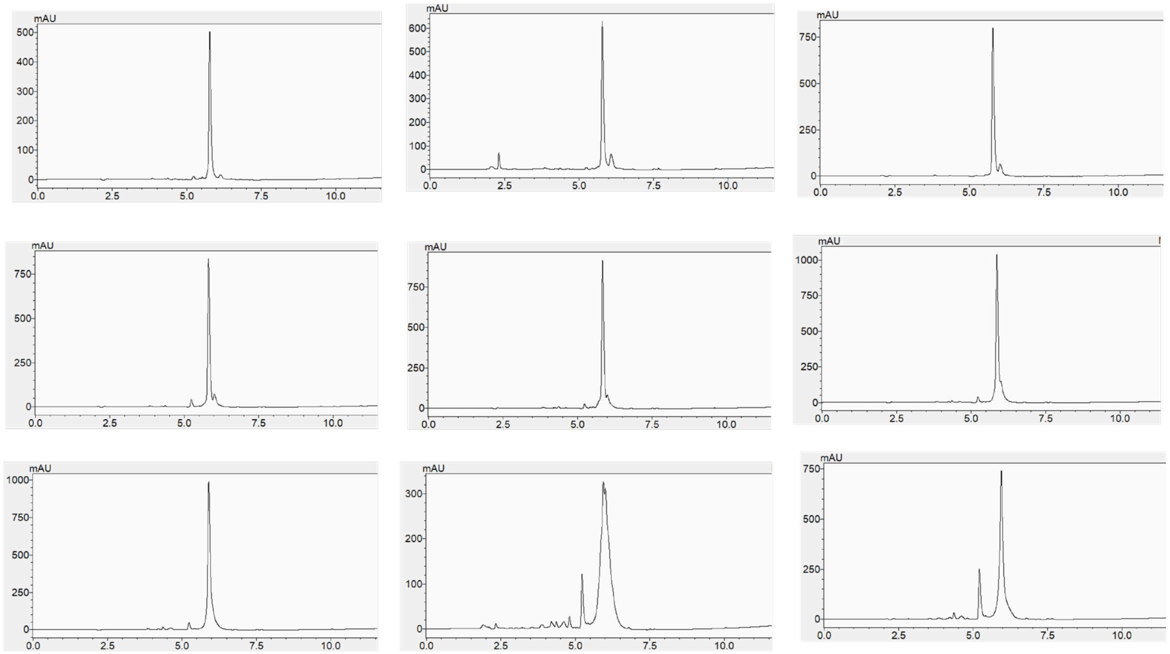

Figure S21. The HPLC results of oligomer OC samples after the bead purification, from n = 3 to n = 11.

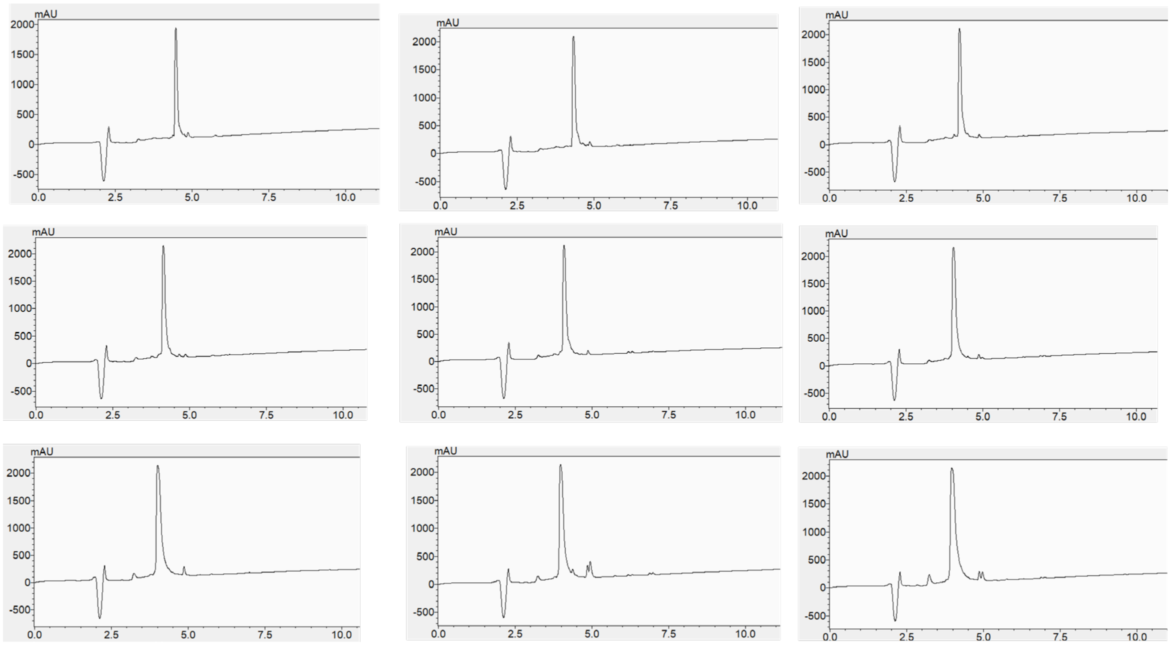

Figure S22. The HPLC results of oligomer OD samples after the bead purification, from n = 3 to n = 11.

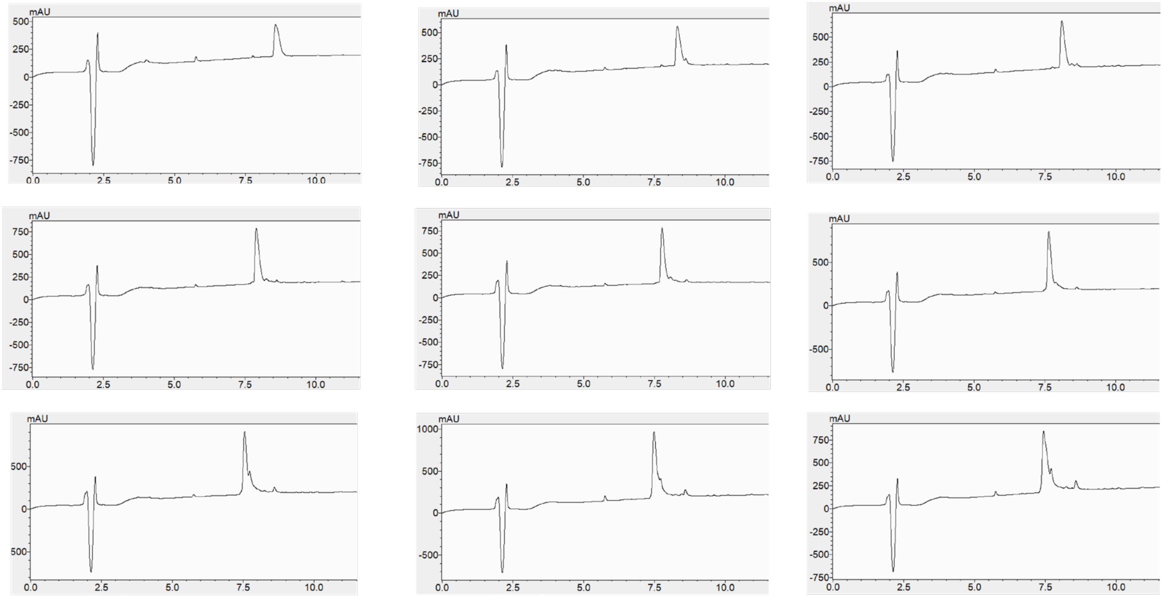

Figure S23. The HPLC results of oligomer OE samples after the bead purification, from n = 3 to n = 11.

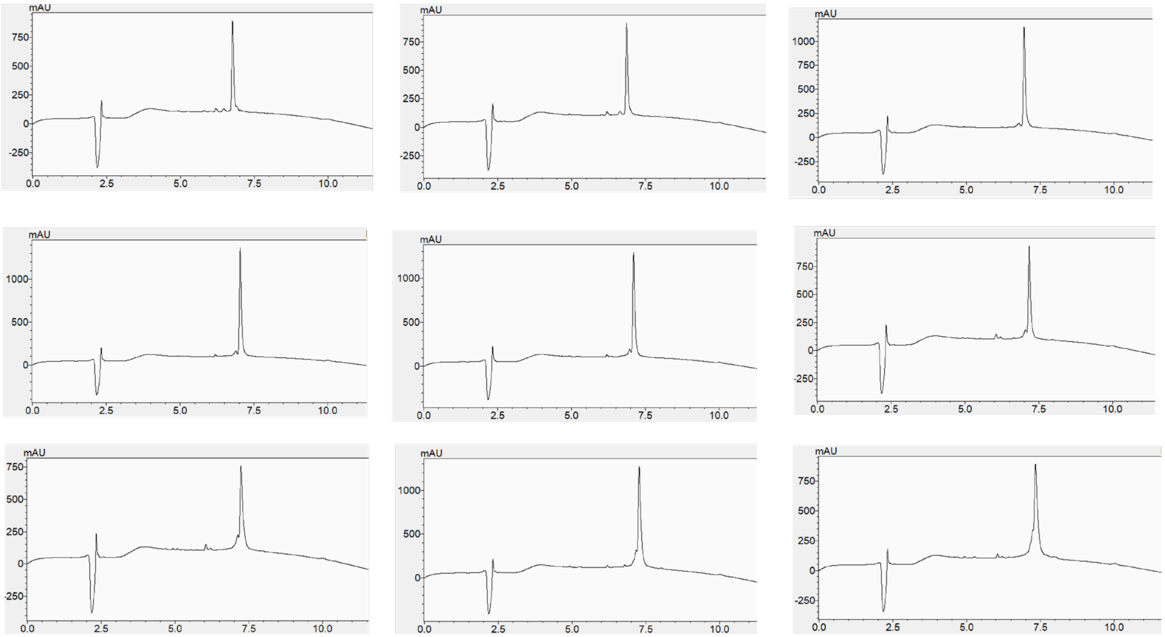

Figure S24. The HPLC results of oligomer OF samples after the bead purification, from n = 3 to n = 11.

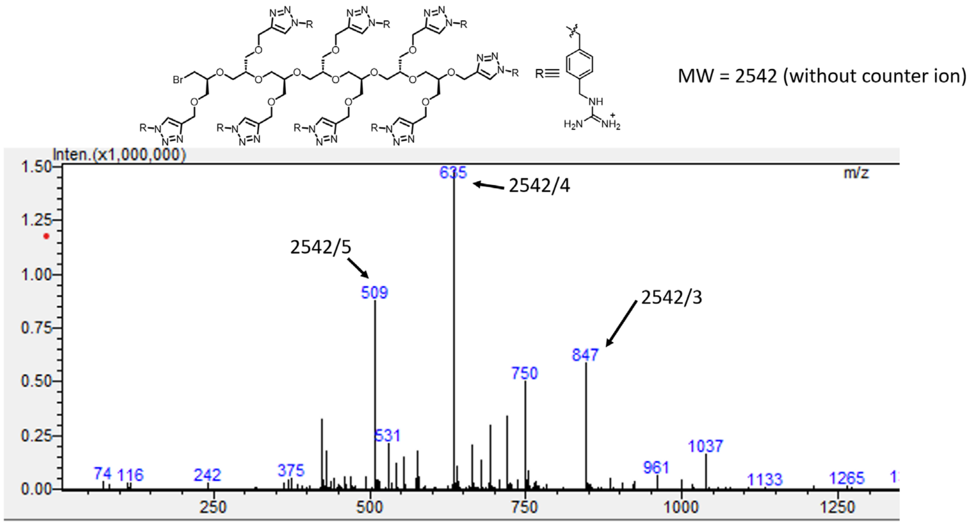

Figure S25. ESI mass spectrum of guanidinium containing oligomer OB(n = 7).

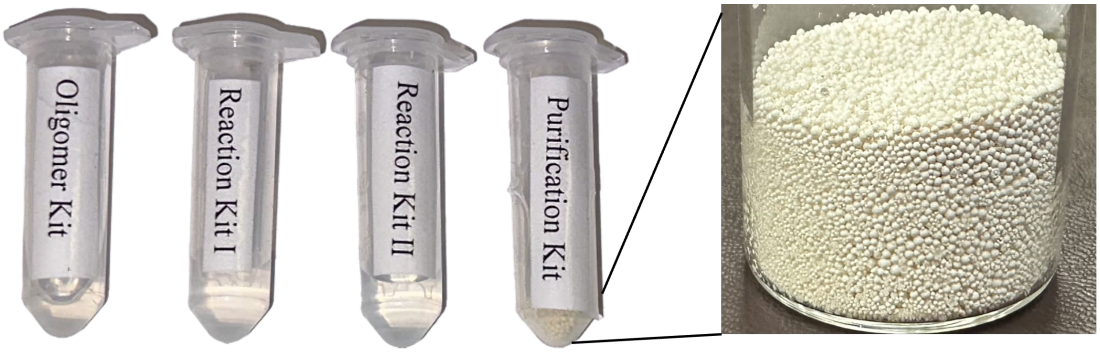

Figure S26. The representative example of the reaction kits for developing discrete oligomers. Oligomer Kit contains one of the discrete alkyne oligomers in ethanol at 20 mM. Reaction Kit I: [azido compound] = 100 mM, [sodium ascorbate] = 2 mM, [BTTAA] = 1 mM in water. Reaction Kit II: [$CuSO_4$] = 1 mM in water. Purification Kit: the purification beads were modified with the DBCO group.

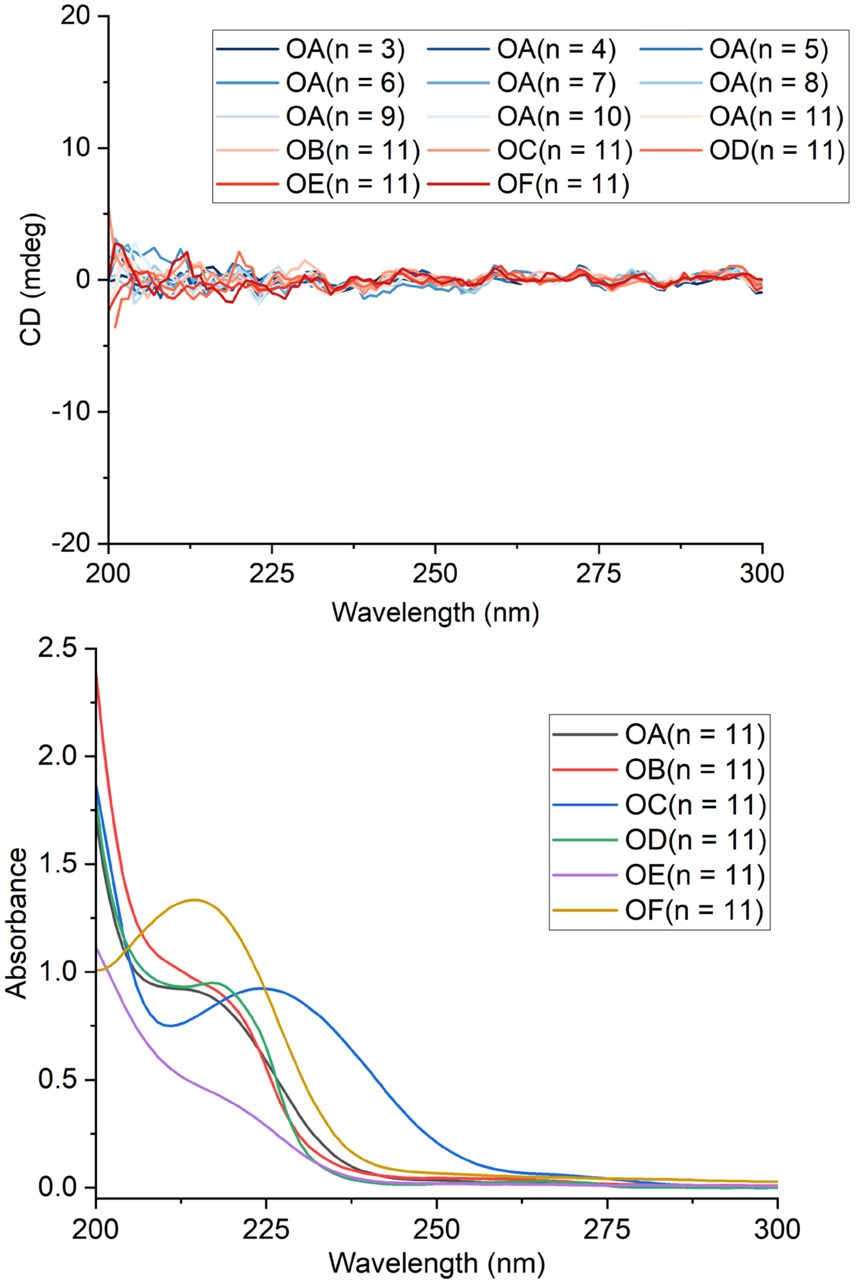

Figure S27. The circular dichroism spectra and UV spectra of the oligomer library in water.

Biological Assays and Results

Antibacterial studies (*E. coli, B. subtilis*)

In a 96-well plate, each well was added 50 µL of Mueller-Hinton broth (MHB) media containing different concentrations of different oligomer samples. Then, 50 µL of bacteria cells in MHB media (OD_{600} = 0.02) was added to each well.

For *E. coli* cells, the final concentrations are:
E. coli OD_{600}= 0.01.
[oligomer] = 0, 2, 4, 8, 16, 32, 64, 128 µM.

For *B. subtilis* cells, the final concentrations are:
B. subtilis OD_{600}= 0.01.
[oligomer] = 0, 1, 2, 4, 8, 16, 32, 64 µM.

The bacteria cells were incubated at 37 °C for 8 h, and the bacteria growth was measured by using a plate reader at 600 nm.

HeLa cell viability studies

In a 96-well plate, each well was added 50 µL of DMEM media (10% FBS added) containing different concentrations of different oligomer samples. Then, 10000 HeLa cells in 50 µL of DMEM media (10% FBS added) were added to each well.

The final concentrations are:
HeLa cells: 10000 cells in 100 µL of DMEM media.
[oligomer] = 0, 2, 4, 8, 16, 32, 64, 128 µM.

The cells were incubated at 37 °C with 5% CO_2 for 24 h, and the cell viability was measured by using MTT assay.

a

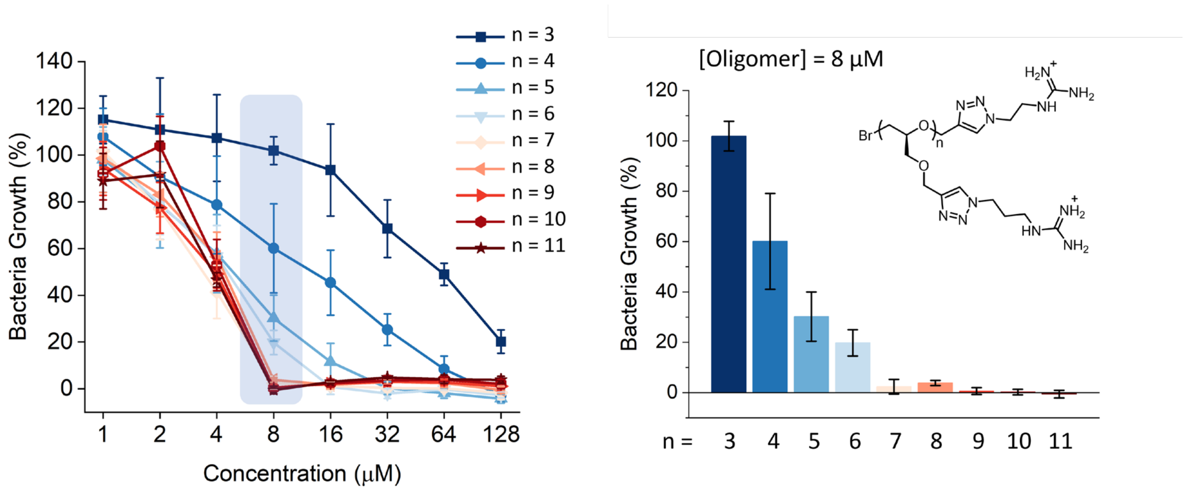

b

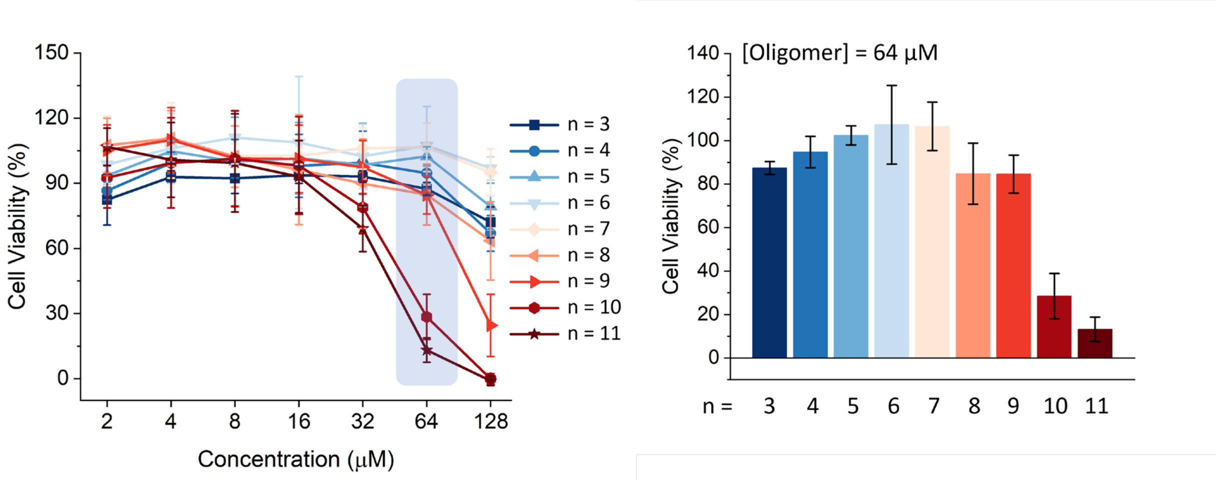

Figure S28. Concentration dependent antibacterial behaviors (**a**) and cytotoxicity (**b**) of the guanidinium functionalized discrete oligomers.

	n = 3	n = 4	n = 5	n = 6	n = 7	n = 8	n = 9	n = 10	n = 11
OA	0.0625	1	4	4	16	4	8	1	4
OB	1	2	4	1	0.25	0.125	0.03125	0.0625	0.03125
OC	0.25	64	64	64	16	16	8	4	16
OD	0.015625	0.0625	4	1	1	4	4	2	2
OE	2	1	1	0.5	0.0625	0.0625	0.0625	0.0625	0.0625
OF	0.003906	0.003906	0.003906	0.003906	0.003906	0.003906	0.003906	0.003906	0.003906

Figure S29. Heat map of the apparent therapeutic index divided by MIC50 (IC50/MIC50^2) of the oligomer library on *E. coli*.

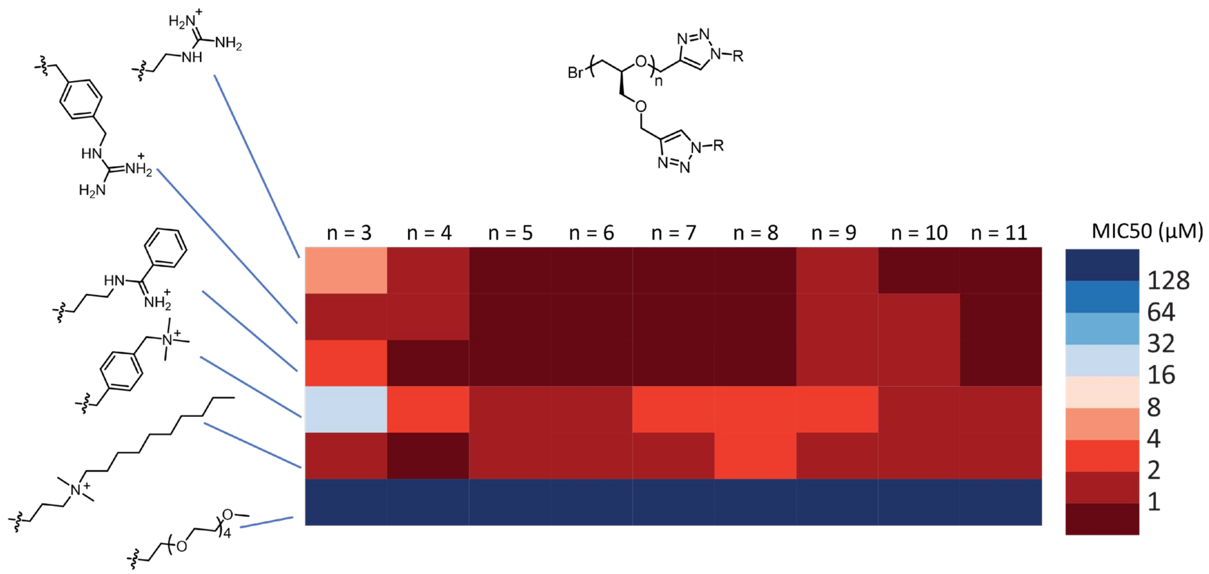

Figure S30. The antibacterial MIC50 summary of *B. subtilis*.

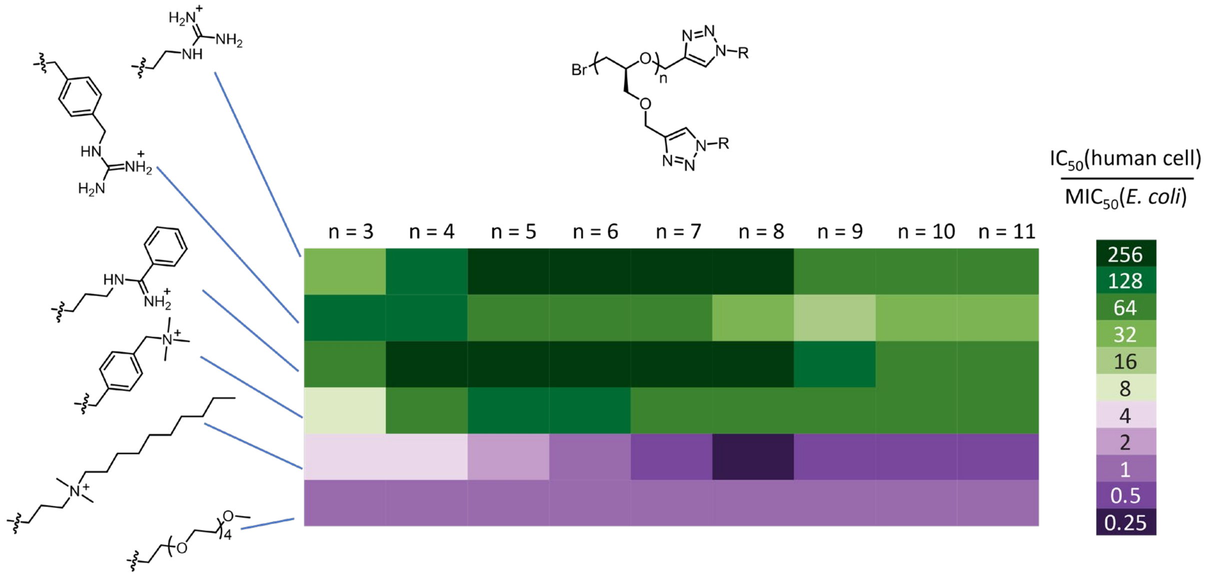

Figure S31. The result summary of IC50 of HeLa cell viability divided by MIC50 of *B. subtilis*.

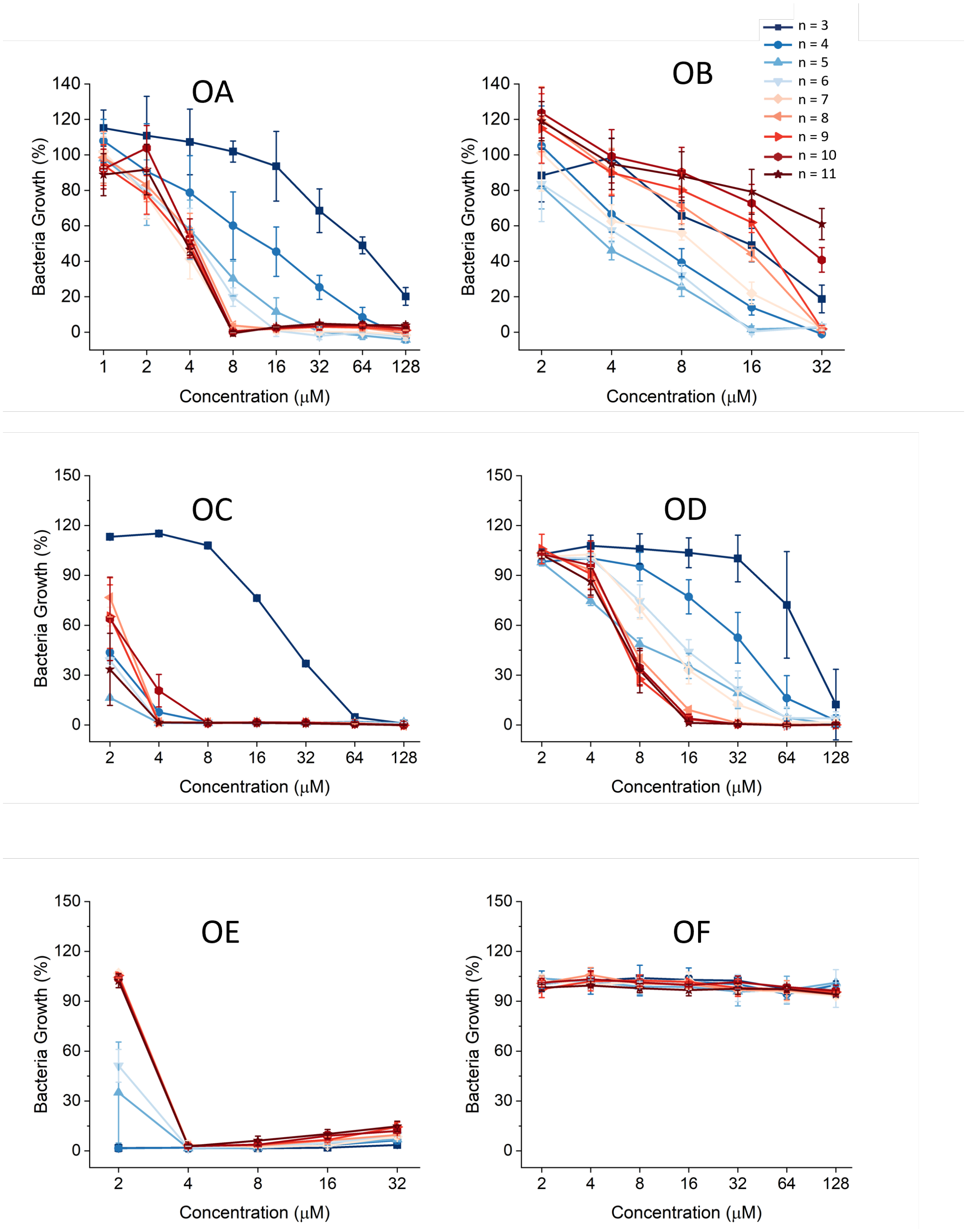

Figure S32. *E. coli* cell growth under different concentrations of different discrete oligomers.

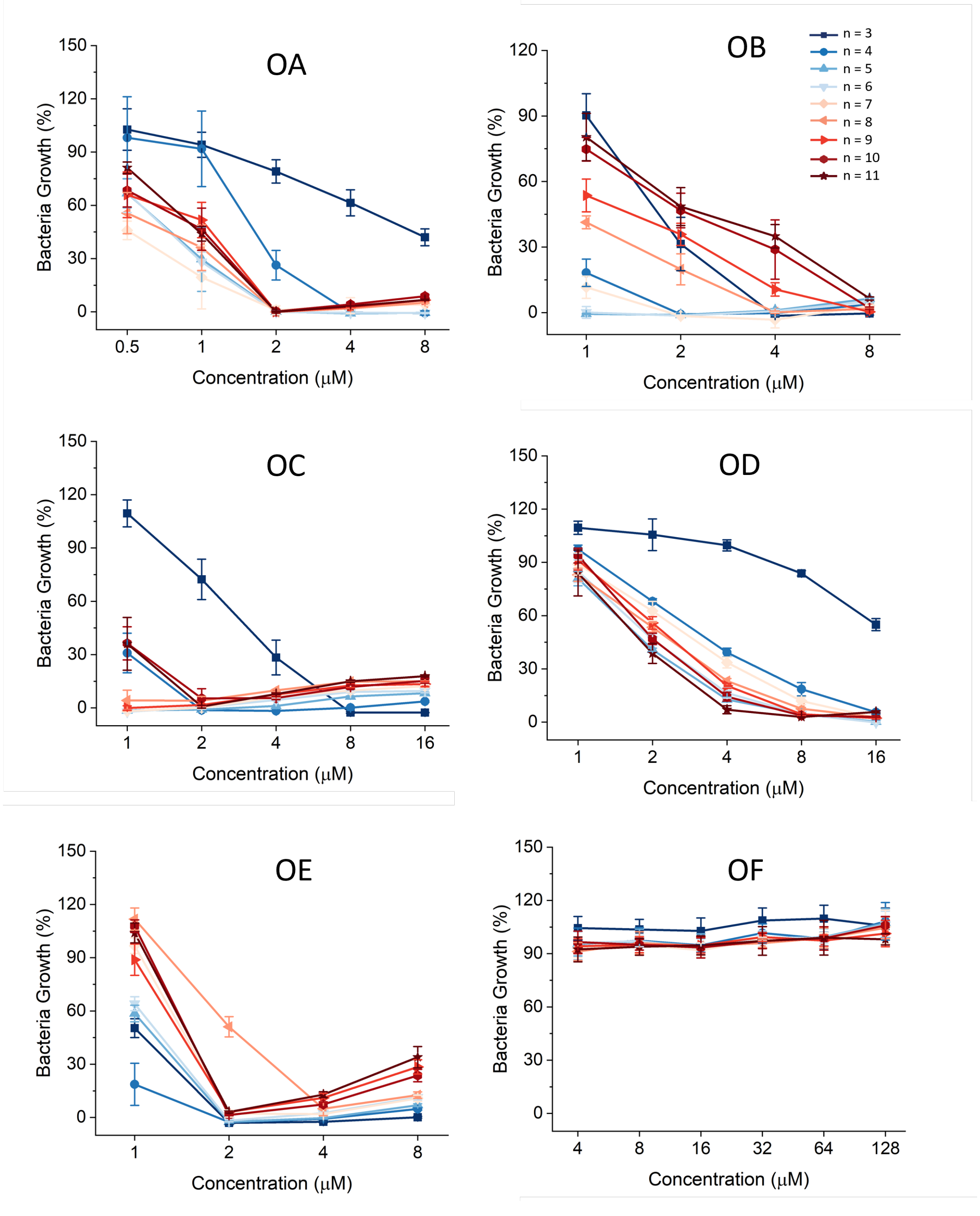

Figure S33. *B. subtilis* cell growth under different concentrations of different discrete oligomers.

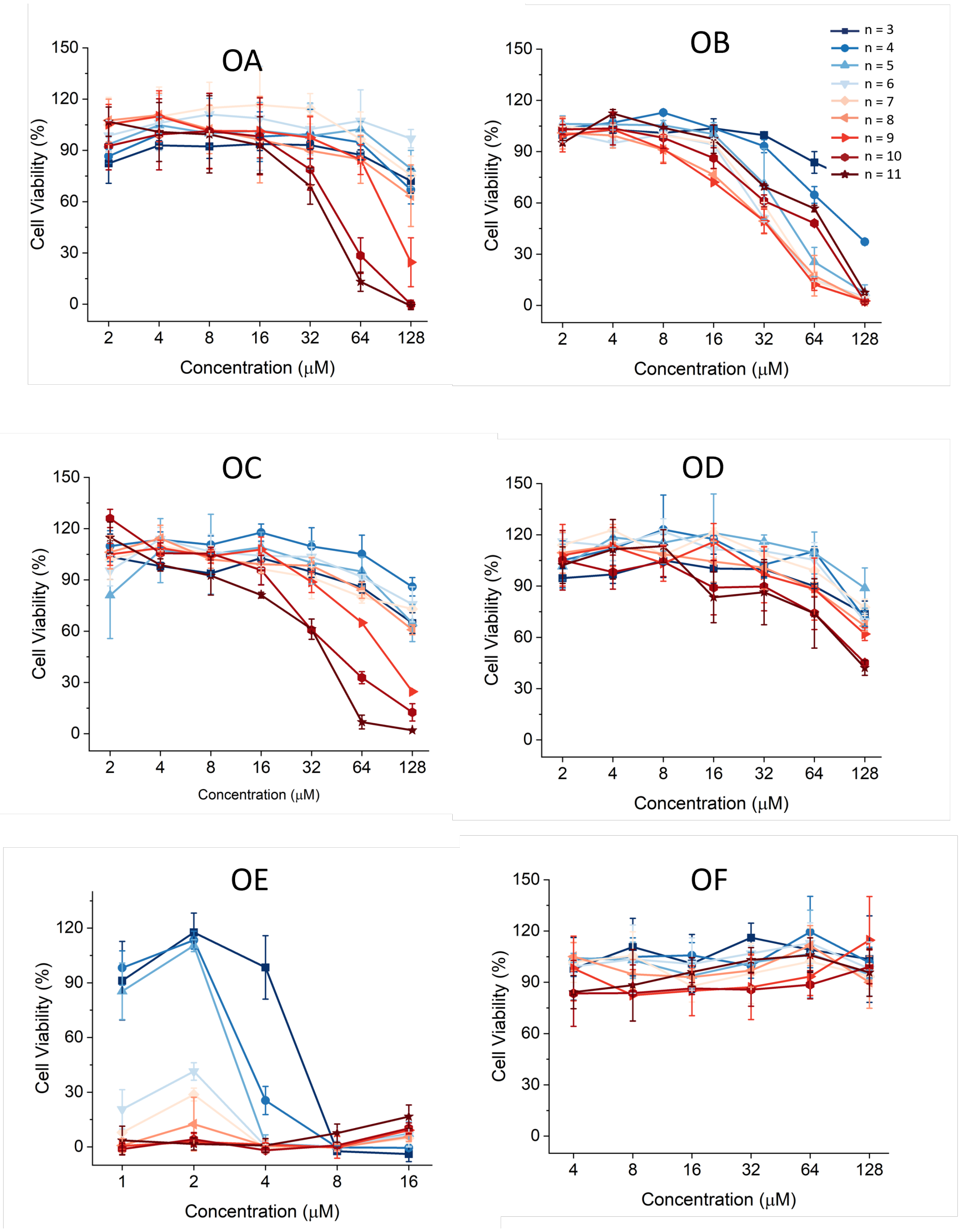

Figure S34. HeLa cell viability with different concentrations of different discrete oligomers.

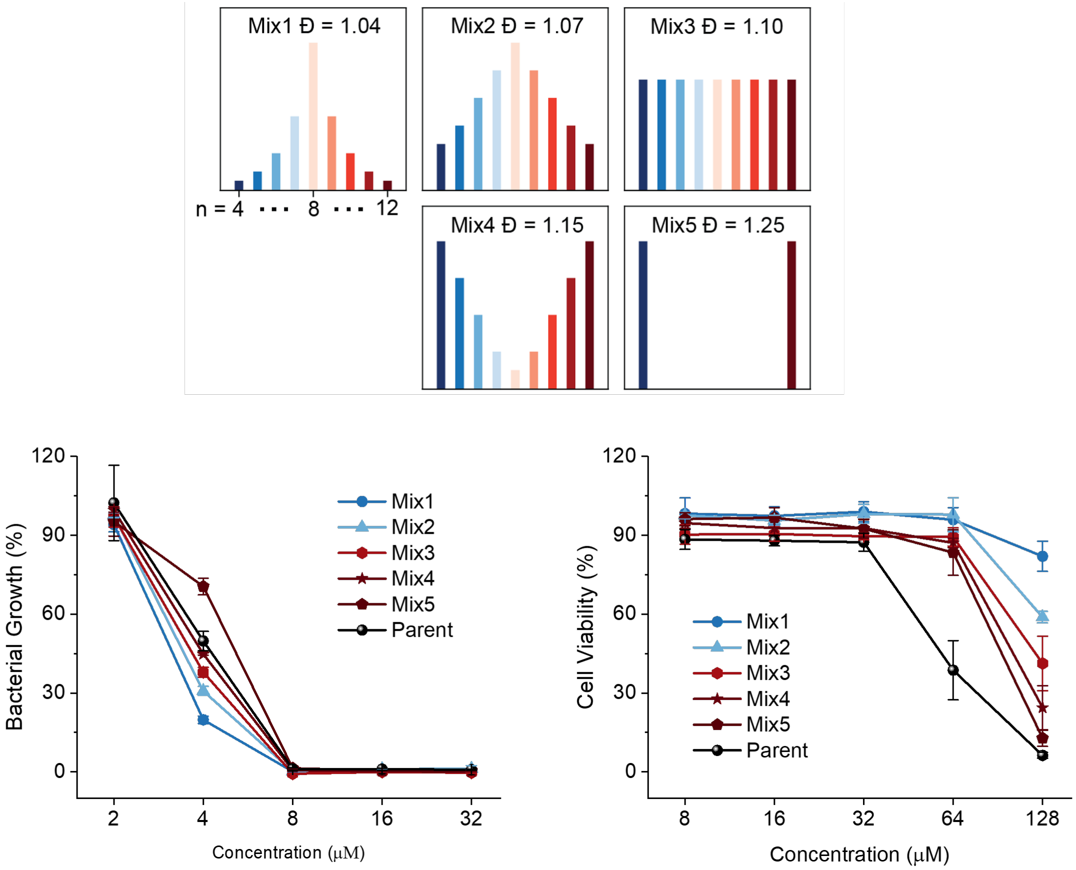

Figure S35. Concentration dependent E.coli growth and HeLa cell viability of artificial formulated mixtures and the unseparated parent sample.

Reference

(1) Bobrin, V. A.; Chen, S.-P.; Grandes Reyes, C. F.; Sun, B.; Ng, C. K.; Kim, Y.; Purcell, D.; Jia, Z.; Gu, W.; Armstrong, J. W.; McAuley, J.; Monteiro, M. J. Water-Borne Nanocoating for Rapid Inactivation of SARS-CoV-2 and Other Viruses. *ACS Nano* **2021**, *15*, 14915-14927.

(2) Kobayakawa, T.; Tsuji, K.; Konno, K.; Himeno, A.; Masuda, A.; Yang, T.; Takahashi, K.; Ishida, Y.; Ohashi, N.; Kuwata, T.; Matsumoto, K.; Yoshimura, K.; Sakawaki, H.; Miura, T.; Harada, S.; Matsushita, S.; Tamamura, H. Hybrids of Small-Molecule CD4 Mimics with Polyethylene Glycol Units as HIV Entry Inhibitors. *J. Med. Chem.* **2021**, *64*, 1481-1496.

(3) McNelles, S. A.; Pantaleo, J. L.; Adronov A. Highly Efficient Multigram Synthesis of Dibenzoazacyclooctyne (DBCO) without Chromatography. *Org*. *Process Res*. *Dev*. **2019**, *23*, 2740–2745
